# Sexual dimorphism and the multi-omic response to exercise training in rat subcutaneous white adipose tissue

**DOI:** 10.1038/s42255-023-00959-9

**Published:** 2024-05-01

**Authors:** Gina M. Many, James A. Sanford, Tyler J. Sagendorf, Zhenxin Hou, Pasquale Nigro, Katie L. Whytock, David Amar, Tiziana Caputo, Nicole R. Gay, David A. Gaul, Michael F. Hirshman, David Jimenez-Morales, Malene E. Lindholm, Michael J. Muehlbauer, Maria Vamvini, Bryan C. Bergman, Facundo M. Fernández, Laurie J. Goodyear, Andrea L. Hevener, Eric A. Ortlund, Lauren M. Sparks, Ashley Xia, Joshua N. Adkins, Sue C. Bodine, Christopher B. Newgard, Simon Schenk, The MoTrPAC Study Group, Jose Juan Almagro Armenteros, Mary Anne S. Amper, Euan Ashley, Aneesh Kumar Asokan, Julian Avila-Pacheco, Dam Bae, Marcas M. Bamman, Nasim Bararpour, Jerry Barnes, Thomas W. Buford, Charles F. Burant, Nicholas P. Carbone, Steven A. Carr, Toby L. Chambers, Clarisa Chavez, Roxanne Chiu, Clary B. Clish, Gary R. Cutter, Surendra Dasari, Courtney Dennis, Charles R. Evans, Facundo M. Fernandez, Nicole Gagne, Yongchao Ge, Bret H. Goodpaster, Marina A. Gritsenko, Joshua R. Hansen, Krista M. Hennig, Kim M. Huffman, Chia-Jui Hung, Chelsea Hutchinson-Bunch, Olga Ilkayeva, Anna A. Ivanova, Pierre M. Jean Beltran, Christopher A. Jin, Maureen T. Kachman, Hasmik Keshishian, William E. Kraus, Ian Lanza, Bridget Lester, Jun Z. Li, Ana K. Lira, Xueyun Liu, Kristal M. Maner-Smith, Sandy May, Matthew R. Monroe, Stephen Montgomery, Ronald J. Moore, Samuel G. Moore, Daniel Nachun, K. Sreekumaran Nair, Venugopalan Nair, Archana Natarajan Raja, Michael D. Nestor, German Nudelman, Vladislav A. Petyuk, Paul D. Piehowski, Hanna Pincas, Wei-Jun Qian, Alexander Raskind, Blake B. Rasmussen, Jessica L. Rooney, Scott Rushing, Mihir Samdarshi, Stuart C. Sealfon, Kevin S. Smith, Gregory R. Smith, Michael Snyder, Cynthia L. Stowe, Jennifer W. Talton, Christopher Teng, Anna Thalacker-Mercer, Russell Tracy, Todd A. Trappe, Mital Vasoya, Nikolai G. Vetr, Elena Volpi, Michael P. Walkup, Martin J. Walsh, Matthew T. Wheeler, Si Wu, Elena Zaslavsky, Navid Zebarjadi, Tiantian Zhang, Bingqing Zhao, Jimmy Zhen

**Affiliations:** 1 Biological Sciences Division, Pacific Northwest National Laboratory, Richland, WA USA; 2 Department of Biochemistry, Emory University School of Medicine, Atlanta, GA USA; 3 Section on Integrative Physiology and Metabolism, Joslin Diabetes Center, Harvard Medical School, Boston, MA USA; 4 Translational Research Institute, AdventHealth, Orlando, FL USA; 5 Division of Cardiovascular Medicine, Department of Medicine, Stanford University, Stanford, CA USA; 6 Department of Genetics, Stanford University, Stanford, CA USA; 7 School of Chemistry and Biochemistry, Georgia Institute of Technology, Atlanta, GA USA; 8 Duke Molecular Physiology Institute and Sarah W. Stedman Nutrition and Metabolism Center, Duke University Medical Center, Durham, NC USA; 9 Division of Endocrinology, Diabetes, and Metabolism, University of Colorado Anschutz Medical Campus, Aurora, CO USA; 10 Division of Endocrinology, Diabetes, and Hypertension, Department of Medicine, University of California, Los Angeles, CA USA; 11 National Institute of Diabetes and Digestive and Kidney Diseases, National Institutes of Health, Bethesda, MD USA; 12 Aging and Metabolism Research Program, Oklahoma Medical Research Foundation, Oklahoma City, OK USA; 13 Department of Internal Medicine, Carver College of Medicine, University of Iowa, Iowa City, IA USA; 14 Department of Orthopaedic Surgery, School of Medicine, University of California San Diego, La Jolla, CA USA; 15 Icahn School of Medicine at Mount Sinai, New York, NY USA; 16 The Mayo Clinic, Rochester, MN USA; 17 Broad Institute of MIT and Harvard, Cambridge, MA USA; 18 The University of Alabama at Birmingham, Birmingham, AL USA; 19 Wake Forest University School of Medicine, Winston-Salem, NC USA; 20 University of Michigan, Ann Arbor, MI USA; 21 Ball State University, Muncie, IN USA; 22 Larner College of Medicine at the University of Vermont, Burlington, VT USA; 23 University of Texas Medical Branch, Galveston, TX USA

**Keywords:** Proteomics, Transcriptomics, Metabolomics, Lipidomics

## Abstract

Subcutaneous white adipose tissue (scWAT) is a dynamic storage and secretory organ that regulates systemic homeostasis, yet the impact of endurance exercise training (ExT) and sex on its molecular landscape is not fully established. Utilizing an integrative multi-omics approach, and leveraging data generated by the Molecular Transducers of Physical Activity Consortium (MoTrPAC), we show profound sexual dimorphism in the scWAT of sedentary rats and in the dynamic response of this tissue to ExT. Specifically, the scWAT of sedentary females displays -omic signatures related to insulin signaling and adipogenesis, whereas the scWAT of sedentary males is enriched in terms related to aerobic metabolism. These sex-specific -omic signatures are preserved or amplified with ExT. Integration of multi-omic analyses with phenotypic measures identifies molecular hubs predicted to drive sexually distinct responses to training. Overall, this study underscores the powerful impact of sex on adipose tissue biology and provides a rich resource to investigate the scWAT response to ExT.

## Abstract (web summary)

Using a multi-omics approach, the authors examine the molecular drivers of sexual dimorphism in the subcutaneous adipose tissue from sedentary and endurance-trained rats. These data provide a valuable resource for adipose tissue-related research.

## Main

ScWAT is a dynamic storage and secretory organ composed of lipid-storing adipocytes and numerous other cell types (for example, immune cells, endothelial cells and mesenchymal cells)^1–3^. Through the release of a diverse array of signaling molecules such as adipokines, cytokines, growth factors and lipid-derived compounds, scWAT impacts multiple biological processes that are critical for maintaining systemic health^1,2,4–6^. Indeed, numerous factors secreted by or residing in scWAT are implicated in the development of lifestyle-related diseases such as obesity, type 2 diabetes, insulin resistance and cardiovascular disease. Accordingly, regulation of scWAT biology, particularly in response to physiological stressors such as exercise, diet and age, is an important area for study.

ExT improves scWAT metabolic flexibility, lipid flux, insulin sensitivity and immune-cell polarization and expansion (factors that are linked to the risk and severity of cardiometabolic diseases^2,4,7–9^). The profound effect of exercise on scWAT is illustrated by the impact of as little as 11 d of voluntary wheel running on the expression of thousands of genes in murine adipose tissue^7^. Despite recent advances in the field of adipocyte biology, the molecular landscape of scWAT remodeling with exercise is not fully understood. This is especially true because most ExT studies focus on animal models of obesity. Therefore, defining training adaptations in the adipose tissue of lean animals provides an important complement to such studies, yielding a more balanced understanding of scWAT remodeling mechanisms.

In humans and rodents, WAT distribution and content varies considerably between sexes, possibly contributing to sexual dimorphism in disease risk^9–12^. Although WAT is one of the most sexually dimorphic tissues^13,14^, few studies account for sex when studying exercise adaptations. While many sex-driven differences may be attributable to sex hormones^9,15–18^, scWAT is also sexually distinct before puberty, which suggests other potential contributors^9,10,15,16^. Despite these interesting and translationally relevant differences in male and female WAT, the molecular hubs orchestrating sexual dimorphism in WAT and its response to ExT remain largely unexplored.

To provide insight into potential molecular transducers of exercise within and across tissues, the Molecular Transducers of Physical Activity Consortium (MoTrPAC) recently published a multi-omic analysis of 18 solid tissues and blood from male and female Fischer 344 (F344) rats that underwent progressive endurance ExT^13^. Notably, this work identified a strong sex-specific response to exercise in multiple tissues, especially in scWAT. Leveraging this dataset, here we perform a deep dive into the sex-specific and temporal multi-omic response of scWAT to 1, 2, 4 or 8 weeks of treadmill ExT in male and female rats. Our approach allows for scWAT-specific temporal resolution of -omic features (annotated proteins, phosphosites, transcripts, lipids and metabolites) and utilizes weighted gene coexpression network analysis (WGCNA) to identify molecular networks driving temporal -omic and phenotypic responses. Using this model, we identify profound differences in male and female scWAT both at rest and in response to training and identify candidate molecular and cellular transducers that drive distinct phenotypes by sex and in the dynamic response to exercise. Further, our manuscript showcases methods to leverage the first publicly available MoTrPAC multi-omics dataset to drive future hypothesis-based research.

## Results

### Sexually dimorphic phenotypic responses to endurance training

**Fig. 1:**
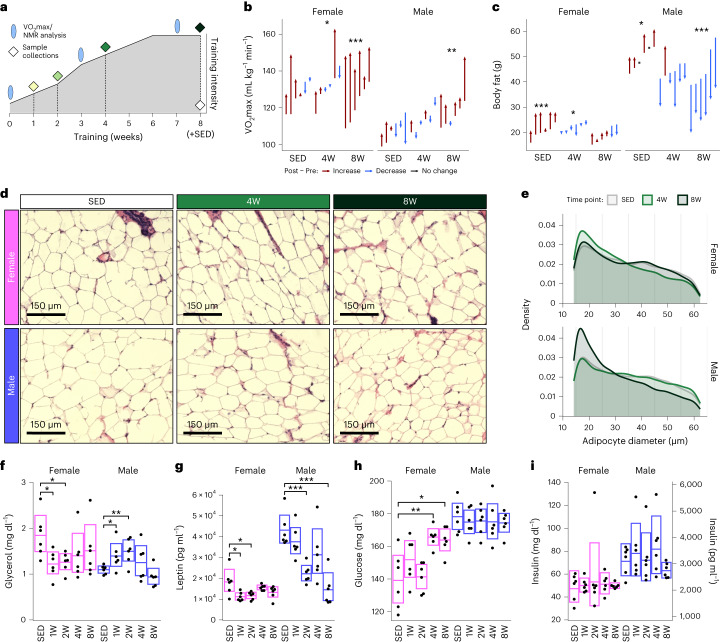
Sexually dimorphic phenotypic responses to endurance training. **a**, Diagram of training protocol. F344 male and female rats underwent a progressive treadmill training protocol. Tissues were collected from animals that completed 1, 2, 4 or 8 weeks of training as well as SED. VO_2_max and NMR body composition analysis was performed as indicated. **b**, Relative VO_2_max values (normalized to NMR lean body mass) recorded pre- and post-training from the animals selected for multi-omics analysis in SED, 4W and 8W time points. 4W, 4-week-trained; 8W, 8-week-trained. **c**, Total fat mass pre- and post-training in SED, 4W and 8W-trained animals, as measured by NMR. Each arrow in **b**,**c** represents a different rat and they span from pre- to post-training values. The rats within each group are arranged in ascending order by their pre-training measure. Paired *t*-tests (*n* = 6 per group) were performed to test for (post − pre) training differences. **d**, Representative histological images (20X magnification) of scWAT from sedentary, 4W and 8W female and male rats. **e**, Adipocyte diameter distributions of histological sections (*n* = 6 rats per group). Tissue sections from each rat were automatically quantified using CellProfiler, with ten fields captured per section for analyses. Dunnett’s test was used to compare each trained group to their sex-matched sedentary controls. **f**–**i**, Measurements and Dunnett’s test results for circulating plasma levels of glycerol (**f**), leptin (**g**), glucose (**h**) and insulin (**i**) from rats selected for multi-omic analysis from each group (*n* = 6). Boxes show the 95% confidence intervals of the group means. The confidence intervals for insulin are just for consistency; time point was not a significant predictor, so timewise comparisons were not performed. In all plots, asterisks indicate statistical significance (*P < 0.05; **P < 0.01; ***P < 0.001); statistical models and tests are described in detail in the Methods. Source data

**Extended Data Fig. 1:**
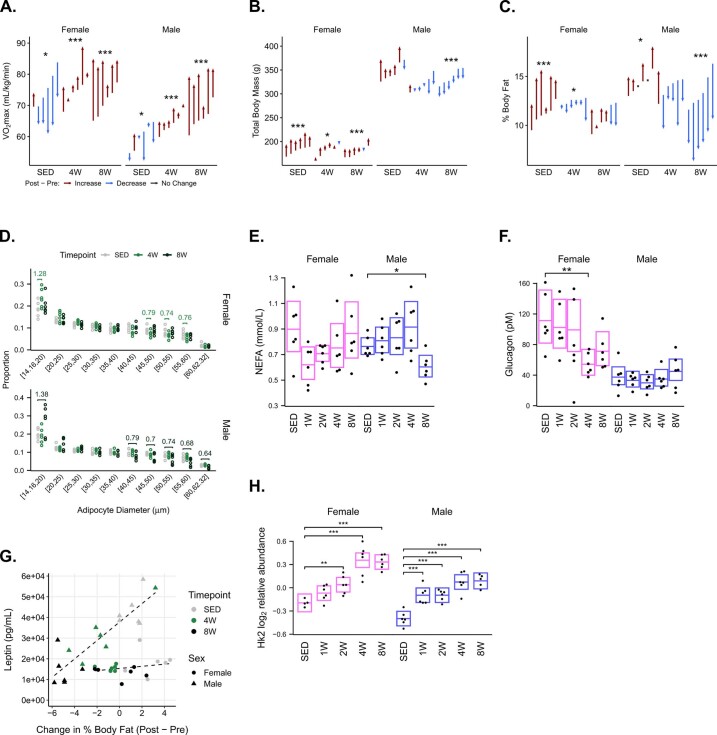
Additional phenotypic data. **a**) Relative VO_2_max values normalized to total body mass from the animals selected for multi-omics analysis. **b–c**) Body mass and percent body fat measurements of animals selected for multi-omics analysis. Arrows represent rats and span from pre- to post-training values. Within each group, they are arranged in ascending order by their pre-training measure. **d**) Proportions of adipocytes from histological analysis with diameters in the indicated ranges for both female and male animals after 4 or 8 weeks of training, or sedentary controls. For comparisons between trained and sedentary groups where statistical significance was reached, the ratio between the group means (trained/sedentary) is specified above the appropriate bin. **e–f**) Additional measures of plasma clinical analytes: non-esterified fatty acids (NEFA) (**e**) and glucagon (**f**). **g**) Correlation between plasma leptin measurements and changes in percent body fat (post vs. pre-training) in each SED, 4 W, and 8 W animal selected for -omics analysis. **h**) Hexokinase 2 protein measures from the gastrocnemius. For (**d–f, h**), trained groups were compared to their sex-matched sedentary controls using Dunnett’s test after fitting a multiple regression model with sex, time point, and their interaction as predictors. Where applicable, the boxes are 95% confidence intervals for the mean of each group, and asterisks indicate a statistically significant difference (*, p < 0.05; **, p < 0.01; ***, p < 0.001) between pre- and post-training measures (**a–c**) or trained and SED groups (**d–f, h**) after adjustment for multiple comparisons.

Beginning at 6 months of age, male and female F344 rats underwent a progressive ExT program consisting of five consecutive days of treadmill running per week for 1, 2, 4 or 8 weeks at a targeted intensity of 70–75% the maximum rate of oxygen consumption (VO_2_max) (Fig. 1a; https://www.motrpac.org/protocols.cfm). This workload was selected for its translational relevance to human studies^19,20^. Sedentary control (SED) rats were age- and sex-matched with the 8-week-trained group. VO_2_max and nuclear magnetic resonance (NMR)-derived body composition were measured at baseline and the start of the final training week in SED, 4- and 8-week-trained rats. Tissues were collected from animals 48 h after the last training session to reduce potential acute impacts of exercise on outcome measures. A subset of 5–6 animals was randomly selected for multi-omic analyses from a larger cohort of 12–20 rats per experimental group (ten combinations of sex and time point). In animals selected for multi-omic analyses, VO_2_max (relative to lean or total body mass) increased similarly in both sexes following 8 weeks of ExT, whereas VO_2_max relative to total body mass decreased significantly in SED rats after 8 weeks of inactivity (Fig. 1b and Extended Data Fig. 1a,b). Body mass and whole-body fat decreased in male rats after 8 weeks of ExT (Fig. 1c and Extended Data Fig. 1b,c). In females, fat mass decreased after 4 weeks of training but returned to pretraining levels at 8 weeks (Fig. 1c and Extended Data Fig. 1b,c). Over the same time period in SED rats, both males and females increased fat mass (Fig. 1c and Extended Data Fig. 1c)^13^. Therefore, 8 weeks of ExT attenuated increases in fat mass in females and reduced fat mass in males.

Given the changes in body composition, we examined scWAT adipocyte size following ExT (Fig. 1d). Analysis of nearly 56,000 total adipocytes revealed differences in size distribution with training in both sexes (Fig. 1e and Supplementary Table 1a,b). Notably, the scWAT of 4-week-trained females showed significant increases in smaller adipocytes (diameter < 20 μm) and corresponding decreases in larger adipocytes (diameter ≥ 45 μm), consistent with the decrease in fat mass in females at the 4-week time point (*P* = 0.02, Extended Data Fig. 1d). Further aligning with changes in fat mass in 8-week-trained females, adipocyte size distribution patterns were similar to those of SED controls (Fig. 1e and Extended Data Fig. 1d), potentially reflecting a compensatory response to preserve scWAT fat stores in female rats with prolonged training. In contrast, scWAT from 8-week-trained male rats showed a significantly higher percentage of small adipocytes and a significantly lower proportion of larger adipocytes compared to SED (Fig. 1e and Extended Data Fig. 1d), indicating a training-induced reduction in scWAT adipocyte size. Thus, despite similar training-induced cardiorespiratory improvements in both sexes, sexually dimorphic ExT responses occurred in overall body composition and scWAT-specific adipocyte morphology.

To further investigate sexual dimorphism in the ExT response, we measured levels of common training-responsive clinical analytes in plasma (Supplementary Table 1c–e). In rats selected for multi-omic analysis, plasma glycerol, a marker of adipose lipolysis, was higher in 1- and 2-week-trained males and lower in 1- and 2-week-trained females relative to SED (*P* = 0.048, *P* = 0.0021, *P* = 0.021 and *P* = 0.014, respectively; Fig. 1f). Non-esterified fatty acids (NEFAs) tended to be higher at early stages of training in male rats but decreased at week 8 (*P* = 0.015), suggestive of triacylglycerol (TAG) mobilization from scWAT in the early training response that could not be sustained as total fat stores depleted with prolonged training (Extended Data Fig. 1e). Consistent with this interpretation, leptin was higher in SED males compared to females and displayed a more robust decrease with training that correlated positively with fat loss (*P* < 0.001; Fig. 1g and Extended Data Fig. 1g). In 4-week-trained females, glucagon decreased, glucose increased and plasma insulin levels did not change (glucagon and glucose *P* < 0.001; Fig. 1h,i and Extended Data Fig. 1f). In contrast, none of these variables were affected by ExT in male rats. Overall, the results highlight sexual dimorphism in systemic and scWAT-specific physiological adaptations to training, several of which serve as indices of enhanced lipid mobilization in males and retention of lipids in adipose stores in females. Sexual dimorphism was also apparent with regard to plasma glucose, glucagon and insulin levels (Fig. 1h,i and Extended Data Fig. 1f), with overall lower plasma insulin levels in females. Despite sexual dimorphism in adipose-associated phenotypes, both sexes displayed similar metabolic adaptations in the skeletal muscle such as hexokinase 2 (HK2) protein abundance (Extended Data Fig. 1h) with ExT; a more comprehensive overview of striated muscle and liver adaptations to training shared in both sexes is detailed by the MoTrPAC Study Group^13^.

### Sedentary rat scWAT displays molecular sexual dimorphism

Considering the sexually divergent phenotypic responses to ExT, we performed a more thorough characterization of the rats selected for multi-omic analysis; scWAT was analyzed at the transcriptomic (*n* = 5), metabolomic (*n* = 5), global proteomic (*n* = 6) and phosphoproteomic (*n* = 6) levels as described in the MoTrPAC study design^20^ using sample-level data available through the MoTrPAC Data Hub (https://motrpac-data.org/). We first investigated molecules and pathways that were differentially expressed between SED males and females.

**Fig. 2:**
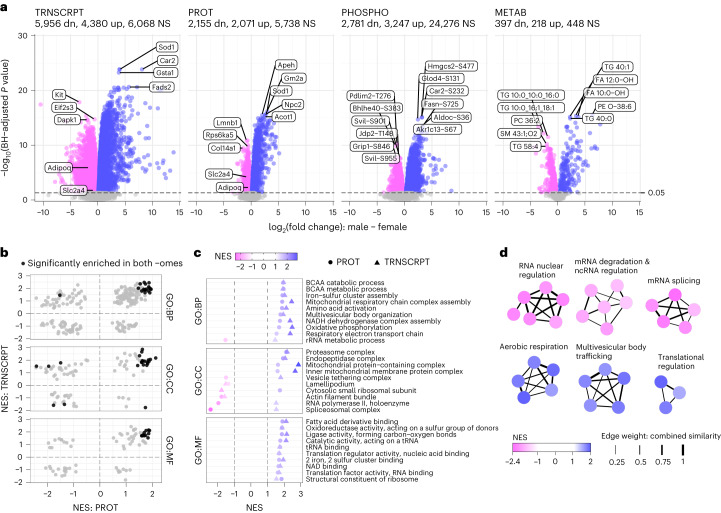
Sexual dimorphism in the molecular landscape of rat scWAT. **a**, Volcano plots displaying the magnitude and significance of changes in transcripts (TRNSCRPT), proteins (PROT), phosphosites (PHOSPHO) and metabolites (METAB) from the differential analysis results comparing male and female SED. Select features are labeled. Blue indicates that a feature was upregulated in males relative to females, pink indicates upregulation in females relative to males and grey indicates no significant change in regulation (BH-adjusted P value ≥ 0.05). dn, downregulated; up, upregulated; NS, not significant. **b**, Scatter-plots of the normalized enrichment scores (NES) for gene sets that were tested in both proteomics and transcriptomics separated by GO. The black points indicate significant (BH-adjusted *P* value < 0.05) enrichment in both -omes. **c**, Scatter-plots of terms that were identified in **b** as being significantly enriched in both -omes in either males (blue) or females (pink). tRNA, transfer RNA; rRNA, ribosomal RNA. **d**, Clusters of top significantly enriched GO-BP terms from the proteomics FGSEA comparison of SED male versus female scWAT. Nodes represent individual GO-BP terms and are colored by the NES; thickness of the edges connecting nodes relates to the proportion of genes in common between the gene sets (see ‘Gene Set Network Diagram’ in Supplementary Methods). ncRNA, noncoding RNA.

Robust sex differences were observed in the abundance of molecules from all -omes; specifically, 10,336 transcripts, 4,226 proteins, 6,028 phosphosites and 615 metabolites showed a statistically significant difference between SED males and females (-ome-wide false discovery rate (FDR) < 0.05), representing 20–60% of all quantified features in each dataset (Fig. 2a and Supplementary Table 2a–d). Top transcripts elevated in SED male scWAT included those related to lipid metabolic processes (*Car2* and *Acss2*) and unsaturated fatty acid (FA) synthesis (*Fads2* and *Fads1*) (Fig. 2a and Supplementary Table 2a). Transcripts that showed higher expression in SED females include molecules involved in growth factor/Akt signaling (*Igf1r*, *Dapk1* and *Kit*), adipogenesis (*Adipoq*) and glucose uptake (*Slc2a4*) (Fig. 2a). At the proteomic level, top differential proteins in males were related to lipid metabolism (Acot1, Hadh and Acbd7), cholesterol transport (Npc2) and ketogenesis (Hmgcl) (Fig. 2a). The scWAT of SED females showed higher abundances of proteins related to nuclear and cellular structural integrity (Lmnb1, Col14a1 and Svil) (Fig. 2a and Supplementary Table 2b). Consistent with transcriptomic data, Adipoq and Slc2a4 (Glut4) protein abundances were higher in female scWAT. The antioxidant Sod1 was elevated in males relative to females at both the messenger RNA and protein levels (Fig. 2a).

Building upon these differential analyses, we performed fast gene set enrichment analysis (FGSEA)^21^ to test for enrichment of Gene Ontology (GO) terms (BP, biological processes; MF, molecular functions; CC, cellular components) (Supplementary Table 3a,b). We also evaluated pathway-level consistency between transcriptomic and proteomic profiles of SED male and female scWAT (Fig. 2b). GO terms related to oxidative metabolism, mitochondrial complex assembly, branched-chain amino acid (BCAA) catabolism, proteasome activation and ribosome subunits were consistently enriched in males at both the transcriptomic and proteomic levels (Fig. 2c). In females, terms including lamellipodium and actin filament bundles were enriched in both -omes (Fig. 2c). When probing the top proteomic networks displaying sexually distinct enrichments, SED males showed enrichment of terms related to aerobic metabolism, multivesicular trafficking (including vesicle ubiquitination-dependent catabolism and sorting) and translational regulation (Fig. 2d). Females displayed upregulation of networks related to messenger RNA (mRNA) nuclear regulation, mRNA splicing and mRNA degradation and noncoding RNA regulatory processes (5.8S RNA maturation and small nucleolar RNA metabolism) at the proteomic level, with immune and developmental terms such as immune effector processes, positive regulation of cytokine production and axon, vasculature and skeletal muscle development terms upregulated at the transcriptomic level (Fig. 2d and Supplementary Table 3a,b).

FGSEA of RefMet chemical subclasses of metabolites^22^ also uncovered sexual dimorphism, with enrichment of amino acid and acyl-CoA species in SED males (Supplementary Table 3e). Female scWAT displayed enrichment of TAG, primarily driven by several long-chain (>40 carbons) TAG species. Some of these metabolomic differences are consistent with enrichment of mitochondrial and amino acid metabolic pathways at the transcriptomic and proteomic levels in males. Taken together, we observed notable differences in the scWAT of SED male and female F344 rats at the transcriptomic, proteomic and metabolomic levels, providing a foundation for understanding the sexually dimorphic response of scWAT to ExT.

### Sex-specific multi-omic scWAT adaptations to ExT

**Fig. 3:**
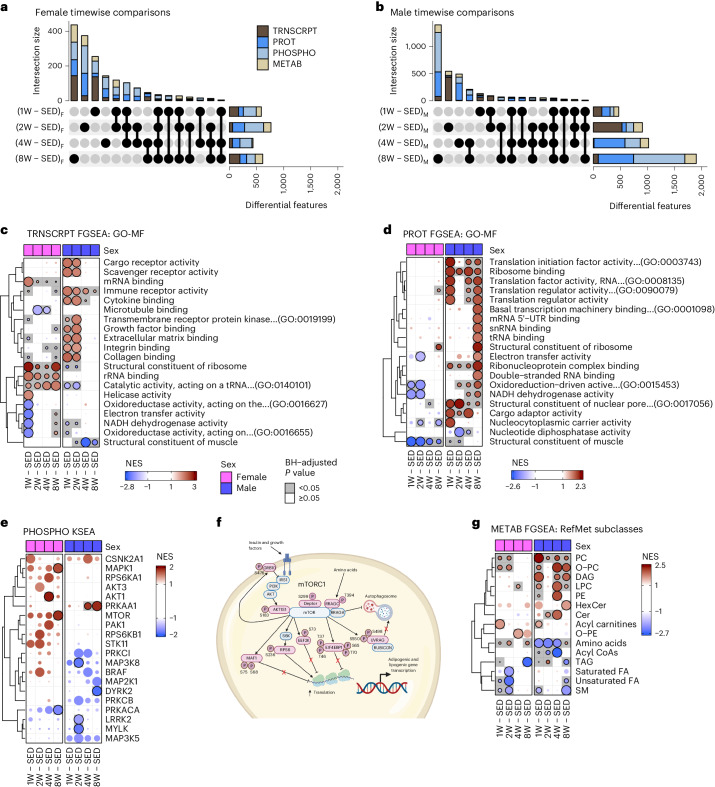
Sex-specific multi-omic scWAT adaptations to ExT. **a**,**b**, UpSet plots of statistically significant (FDR < 0.05) transcripts, proteins, phosphosites and metabolites from each comparison between trained and sex-matched sedentary control female (**a**) and male (**b**) rats. **c**,**d**, Top MF from the GO database that are most significantly enriched in any of the eight comparisons from the transcriptomics (**c**) or proteomics (**d**) FGSEA results. Circles are colored by the NES and scaled by row so that the most significant comparison is of maximum area (‘Fast Gene Set Enrichment Analysis’ Supplementary Methods). UTR, untranslated region. **e**, Inferred activity of the indicated kinases in each of the trained groups versus sex-matched sedentary controls from KSEA of the phosphoproteomics differential analysis results. **f**, Schematic of phosphosites (in pink) driving mTOR enrichment in 8W-trained females (diagram created with BioRender.com). **g**, RefMet chemical subclasses that are significantly enriched in at least one of the eight comparisons according to the metabolomics FGSEA results.

Next, sample-level datasets were analyzed to identify differentially expressed features at each training time point by sex (for example, 1-week-trained females versus SED females; Fig. 3a,b and Supplementary Table 4a–d). In general, female scWAT had a similar number of differential -omic features at all four ExT time points, whereas the number of differentially expressed features increased with training duration in males (Fig. 3a,b).

#### Transcriptomics

**Extended Data Fig. 2:**
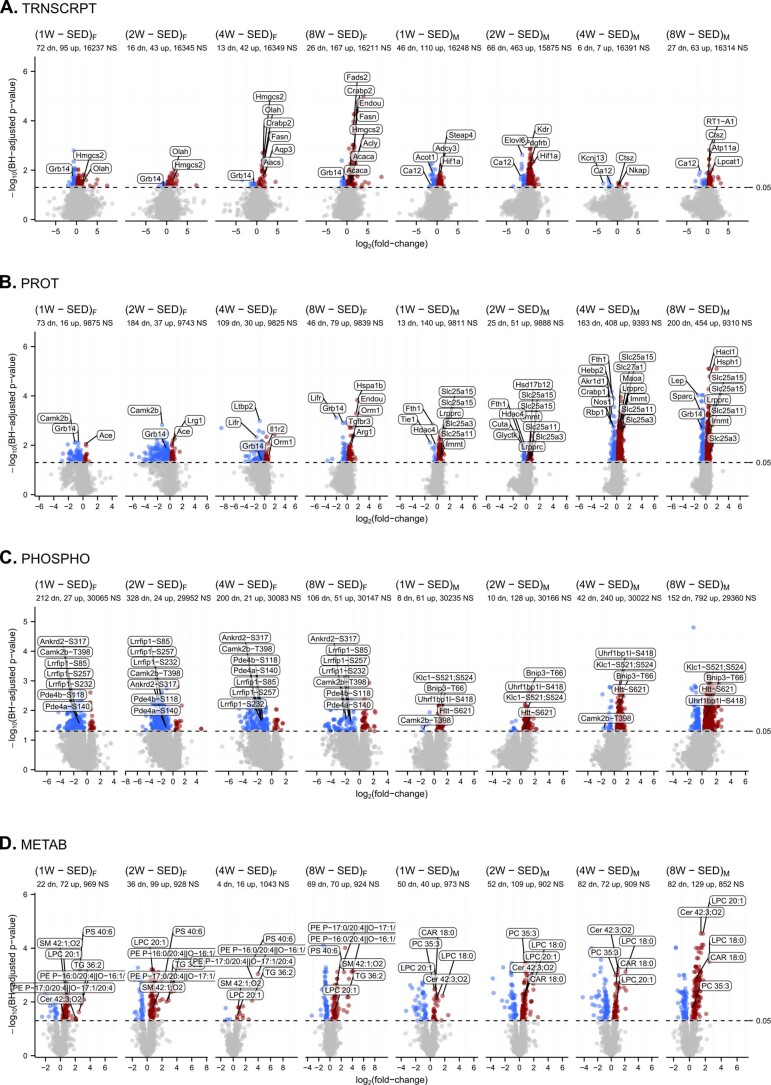
Training response differential expression analysis: volcano plots. **a–d**) Volcano plots displaying the magnitude and significance of comparisons of each trained group against sex-matched sedentary controls in transcriptomics (**a**), proteomics (**b**), phosphoproteomics (**c**), and metabolomics (**d**) datasets. Features that are differentially expressed in all female or all male comparisons are labeled and arranged from top to bottom by most to least significant and along the x-axis according to ranked magnitude of log_2_ fold-change if there was sufficient space. For metabolomics (**d**) the 5 metabolites that are most significant, on average, across all female or male comparisons are labeled since specific metabolites are not mentioned in the differential analysis results.

**Extended Data Fig. 3:**
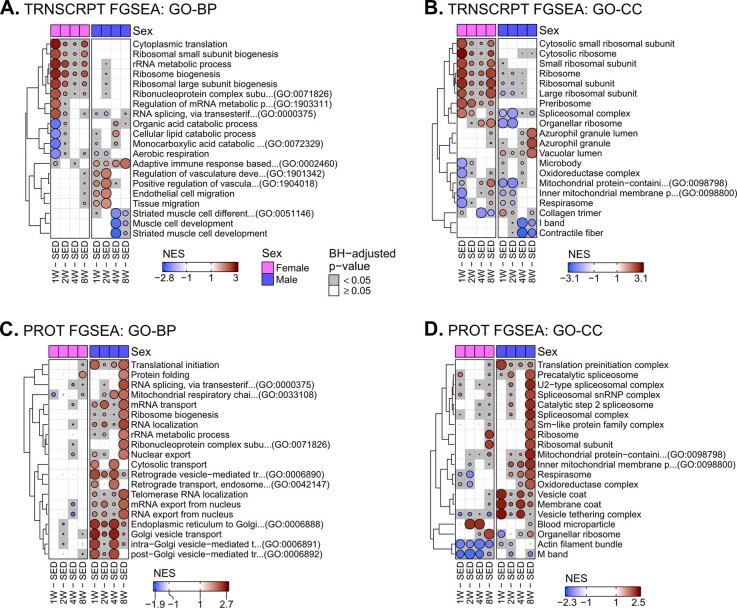
Additional FGSEA heatmaps from training response differential expression analysis. **a–b)** Top biological process (GO-BP) (**a**) or cellular component (GO-CC) (**b**) terms from the Gene Ontology database that are most significantly enriched (FDR < 0.05) in any of the 8 comparisons from transcriptomics FGSEA results. **c–d)** Top GO-BP (**c**) or GO-CC (**d**) terms that are most significantly enriched (FDR < 0.05) in any of the 8 comparisons from proteomics FGSEA results.

ExT induced distinct temporal patterns of differentially regulated transcripts, representing a combined 788 and 474 differentially expressed transcripts across all time points in males and females, respectively (FDR < 0.05) (Supplementary Table 4a). The greatest number of differential transcripts were observed in 1- and 8-week-trained females (167 and 193 transcripts, respectively) and 2-week-trained males (529 transcripts) (Fig. 3a,b and Extended Data Fig. 2a). Six transcripts displayed differential expression at all training time points in females. Notably, *Grb14*, a negative modulator of insulin signaling^23^, was downregulated with ExT, whereas *Olah*, a medium-chain (MC) FA thioesterase and *Hmgcs2*, a ketogenic enzyme with lipogenic roles^24^, were increased at all training time points in females. The only transcript that displayed differential expression at all training time points in males was the hypoxia-inducible carbonic anhydrase (*Ca12*)^25^, which was downregulated. FGSEA further highlighted sex differences in the progressive transcriptomic response to ExT (Fig. 3c, Extended Data Fig. 3a,b and Supplementary Table 5a). After 1 and 2 weeks of ExT, male scWAT was positively enriched for terms related to immune receptor activity and binding of cytokines, growth factors, extracellular matrix, integrins and collagens (Fig. 3c). Enrichment of these pathways was driven by genes involved in tissue stress (*Hif1a*, *Tlr4*, *C4b* and *Nlrp3*), tissue remodeling (*Itgb1*, *Timp3*, *Lifr*, *Pten* and *Fgfr1*) and angiogenesis (*Kdr*, *Flt1*, *Pdgfb* and *Vegfa*) (Fig. 3c and Extended Data Fig. 3a). Enrichment of the GO-MF cytokine binding and immune receptor activity pathways was driven by transcripts related to T helper 17 (T_H_17) cell activation (*Cd4*, *Il17ra*, *Tgfbr2*, *Il6st* and *Ctsl*) in male scWAT at 2 weeks. Females displayed early enrichment of terms related to transcriptional/translational regulation that generally peaked at 1 week and remained enriched throughout training (Fig. 3c and Extended Data Fig. 3a,b). Both males and females displayed an early (1 week) adaptive immune response that was attenuated at later time points in females but remained positively enriched in males throughout ExT (Extended Data Fig. 3a). Terms related to oxidative phosphorylation (OXPHOS) activity decreased in both sexes at 1 week but recovered by 2 weeks in males and 4 weeks in females (Fig. 3c). In 8-week-trained rats, terms related to the adaptive immune system, immune receptor activity, azurophil granules and vacuolar lumen were enriched in males, whereas transcription and translation regulatory processes, aerobic respiration and azurophil granules were enriched in 8-week-trained females (Fig. 3c and Extended Data Fig. 3b).

#### Proteomics

There was a robust change in the scWAT proteome in male rats that increased with training duration (654 differentially expressed proteins at 8 weeks), whereas the response in females was much smaller, with most differences occurring after 2 weeks (221 differential proteins) (Fig. 3a,b, Extended Data Fig. 2b and Supplementary Table 4b). In males, 18 proteins were differentially abundant at all time points, most of which were related to mitochondrial function (Maip, Atp6v0d1 and Immt), mitochondrial solute transport (Slc25a11, Slc25a3 and Slc25a15) and vesicle transport (Tmed2 and Sec61a1). Lrpprc, a protein that regulates transcription of mitochondrial genes to promote lipid oxidation^26^, increased throughout training in males. Slc25a15, a mitochondrial ornithine carrier that promotes arginine synthesis, was also elevated throughout training in male scWAT. Arginine promotes lipolysis partially through Ampk activation^27^. FGSEA revealed a robust increase in GO terms related to OXPHOS and mitochondria-specific ribosome biogenesis in male scWAT starting at 2 weeks of ExT (Fig. 3d, Extended Data Fig. 3c and Supplementary Table 5b). Males also displayed enrichment of terms related to vesicle transport and ribosome activity and biogenesis throughout training (Fig. 3d and Extended Data Fig. 3c,d). Females displayed differential abundance of five proteins at all four time points. Consistent with transcriptomic profiling, the insulin signaling repressor Grb14 (ref. ^23^) decreased in females at all four time points. Further, 4 or 8 weeks of training in females increased expression of anti-inflammatory and insulin-sensitizing Orm1 (ref. ^28^) (Extended Data Fig. 2), suggesting candidate mechanisms by which training improves scWAT insulin signaling in lean females in the absence of fat loss. In contrast to males, female scWAT displayed negative enrichment of terms related to mitochondrial processes at 1 and 2 weeks, with modest enrichment of mitochondrial-related terms at 8 weeks (Extended Data Fig. 3c,d). Both sexes displayed enrichment of terms related to transcription and translation at 8 weeks, with a more robust proteomic than transcriptomic response in males (Fig. 3d and Extended Data Fig. 3c,d).

#### Phosphoproteomics

Several hundred protein phosphorylation sites were differentially regulated with ExT, with sexually dimorphic temporal patterns similar to proteomic ExT responses (Supplementary Table 4c). In general, males displayed a more robust phosphoproteomic response following 4 and 8 weeks of ExT (Fig. 3a,b and Extended Data Fig. 2c). Females modulated 14 phosphoproteins at all four training time points, including decreased phosphorylation of proteins related to inflammatory responses (Pde4 a–b and Lrrfip1-S85) and Camk2b-T398, a negative regulator of adipocyte insulin signaling^29^. Males exhibited consistent modulation of the phosphorylation of 15 proteins throughout ExT, including increased phosphorylation of proteins regulating vesicle transport (Htt-S621, Klc1-S521/S524 and Uhrf1bp1l-S418) and autophagy (Bnip3-T66 and Ulf1-S458). To allow for inference of kinase activity based on changes in phosphorylation of known substrates, we utilized curated kinase–substrate relationship data from PhosphoSitePlus (PSP)^30^ to conduct kinase–substrate enrichment analysis (KSEA) (Fig. 3e and Supplementary Table 5d). The results of this analysis showed decreased activity of MAP3K8, LRRK2 and MYLK kinases in 2-week-trained males (Fig. 3e), whereas activity of the lipolysis-inducing kinase PRKAA1 (AMPK1ɑ1) increased in 4- and 8-week-trained males. DYRK2 activity decreased at 8 weeks in males (Fig. 3e), which was largely driven by changes in CARHSP1 phosphorylation (S30, S32 and S41). In females, AKT1 activity increased at 4 weeks before subsiding by week 8 when MAPK1 and mTOR activity increased. Given the well-defined role of mTORC1 in anabolic signaling, including in adipose tissue^31^, coupled with the observed downregulation of proteins and phosphoproteins associated with insulin signaling repression (for example Grb14 and Camk2b) in female scWAT, we sought to characterize the phosphoproteins indicative of enriched mTOR activity. Increases in mTOR activity are supported by phosphorylation of the insulin-dependent RAGC-T394 phosphosite^32^, RPS6-S236, EIF4EBP1 at S65 and T70, AKT1S1 (PRAS40) at S183 and MAF1 at S68 and S75; a graphical illustration of phosphosites driving mTOR enrichment is presented in Fig. 3f to exemplify how this dataset may be used to drive mechanistic research. In 4-week-trained females, phosphosites driving AKT1 enrichment included the AKT-dependent inactivation site (S253) of catabolic FOXO3 and two sites (S425 and T447) near the activation site of the FA synthesis protein ATP citrate lyase (ACLY)^33^. In adipocytes, ACLY promotes Glut4 expression and de novo lipogenesis in a manner that is more pronounced in females^34^. Together with findings from our differential -omic analyses, these data suggest enrichment in anabolic insulin/AKT/mTORC1 as a candidate mechanism to promote scWAT insulin sensitivity and lipid storage/recycling in females^31^.

#### Metabolomics

**Extended Data Fig. 4:**
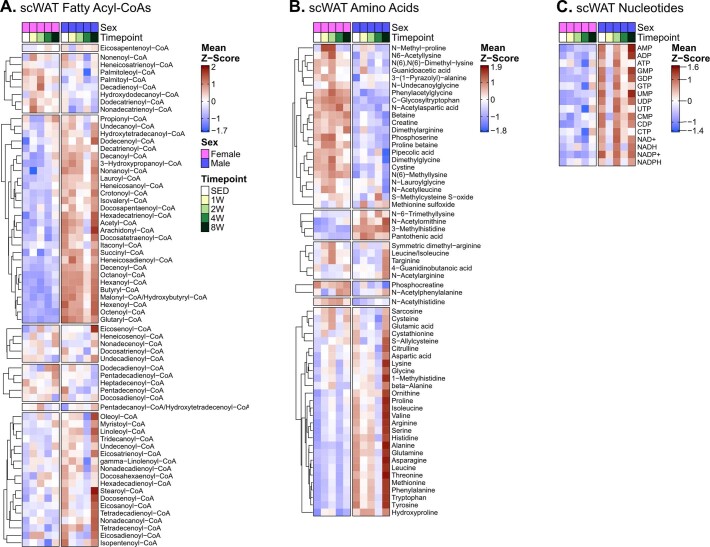
Metabolomic Visualizations. **a–c**) scWAT metabolome heatmaps displaying acyl-CoAs (**a**), amino acids (**b**), and nucleotides (**c**). Sample-level values were standardized before calculating the mean of each group to better observe patterns.

We employed a comprehensive suite of metabolomics technologies to characterize changes in the scWAT metabolome/lipidome with ExT. This included non-targeted methods, as well as quantitative targeted methods focused on groups of chemically related analytes such as amino acids, acylcarnitines and nucleotides^20^. Overall, the scWAT metabolome displayed the least sexually dimorphic ExT response, with the most differentially regulated metabolites appearing at 2 and 8 weeks in both sexes (2 weeks, 135 and 161 differential metabolites in females and males, respectively; 8 weeks, 139 and 211 differential metabolites) (Fig. 3a,b, Extended Data Fig. 2d and Supplementary Table 4d). The 4-week-trained time point displayed the most sexual dimorphism with 154 differentially regulated metabolites in males and only 20 in females. In both sexes, FGSEA of RefMet metabolite subclasses^22^ revealed positive enrichment of phosphatidylcholine (PC), ether phosphatidylcholines (O-PC) and acylcarnitine species in 1-week-trained rats, followed by negative enrichment of TAGs at 8 weeks (Fig. 3g and Supplementary Table 5e). Females displayed a prolonged early enrichment of PC and O-PC phospholipid species, along with a negative enrichment of sphingomyelin (SM) and unsaturated FAs at 1 and 2 weeks. Males showed positive enrichment of O-PC, diacylglycerols (DAGs) and lysophosphatidylcholines (LPCs) at 1, 4 and 8 weeks of ExT and PC at all four ExT time points (Fig. 3g). Males additionally displayed an increase in phosphatidylethanolamine (PE) at 4 weeks that subsided by week 8 (Fig. 3g). PE and PC are the major phospholipid species present in the phospholipid monolayer of the lipid droplet (LD). The increase of PC relative to PE in 8-week-trained males is consistent with our observation of reduced adipocyte size, which suggests that males display attenuated LD formation after 8 weeks^5,35^ and is supported by phenotypic changes in adipocyte size distribution and total fat mass. Notably, acyl-CoAs, which were elevated in the scWAT of SED males, decreased in 4-week-trained males (Fig. 3g and Extended Data Fig. 4a), whereas amino acids increased sharply at 8 weeks, further supporting increased lipid utilization and a switch to amino acid metabolism with ExT in males. This interpretation is also consistent with the surge in mono- and di-nucleotide phosphate species and reduction in nucleotide triphosphates and increased AMP:ATP ratio corresponding to scWAT fatty acyl-CoA abundance at 8 weeks in males (Extended Data Fig. 4c). Conversely, females displayed positive enrichment of amino acids at 1, 2 and 8 weeks (Fig. 3g and Extended Data Fig. 4b).

#### Sexual dimorphism in the temporal response to ExT

**Extended Data Fig. 5:**
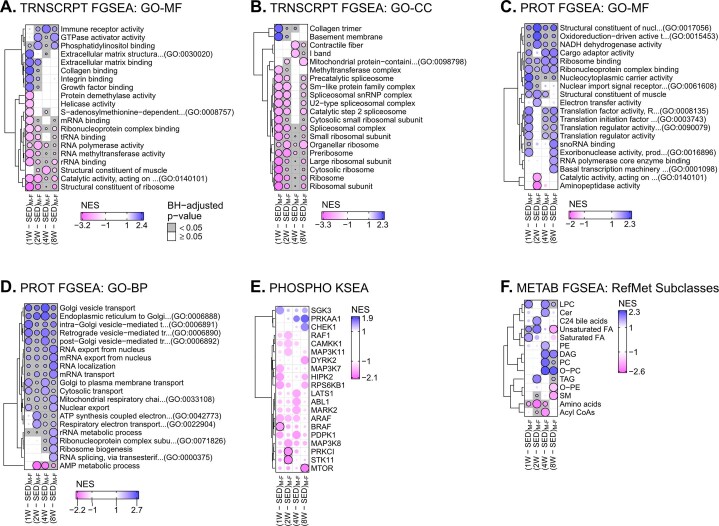
Comparison of training response across sexes. **a–b)** Top molecular function (GO-MF) (**a**) or cellular component (GO-CC) (**b**) terms from the Gene Ontology database that are most significantly enriched (FDR < 0.05) in any of the 4 comparisons of the male and female training responses from the transcriptomics FGSEA results. **c–d)** Top GO-MF (**c**) or GO-BP (**d**) terms that are most significantly enriched (FDR < 0.05) in any of the 4 comparisons of the male and female training responses from the proteomics FGSEA results. **e)** Top kinases that are most significantly enriched (FDR < 0.05) in any of the 4 comparisons of the male and female training responses from the phosphoproteomics KSEA results. **f)** Top RefMet chemical subclasses that are most significantly enriched (FDR < 0.05) in any of the 4 comparisons of the male and female training responses from the metabolomics FGSEA results.

We next examined differences between sexes in response to progressive ExT. To this end, we compared the male ExT response to the female response at each time point (for example (males 1-week − males SED) − (females 1-week − females SED)), an analysis that reveals differences in the magnitude and/or direction of training responses between sexes (Supplementary Table 6a–d). At the transcriptomic level, these analyses uncovered pathways related to immune-receptor activation, GTPase activation and transcriptional/translational regulation as responding most differently to ExT between the sexes (Extended Data Fig. 5a,b and Supplementary Table 7a). At the proteomic level, differences were observed in pathways related to mitochondrial activity, vesicle transport and transcriptional/translational processes (Extended Data Fig. 5c,d and Supplementary Table 7b). The strongest phosphoproteomic differences were predicted increases in the kinase activity of mTOR in 8-week-trained females and PRKAA1 (AMPKɑ1) in 4- and 8-week-trained males (Extended Data Fig. 5e and Supplementary Table 7c). The metabolomic response to ExT displayed sexual divergence in amino acid, acyl-CoA, DAG, PC, O-PC and LPC abundance (Extended Data Fig. 5f), with females displaying enrichment in amino acids in the early training response, which is suggestive of an early anabolic response to training (Supplementary Table 7d).

### Integrative phenotypic–omic responses to ExT

**Fig. 4:**
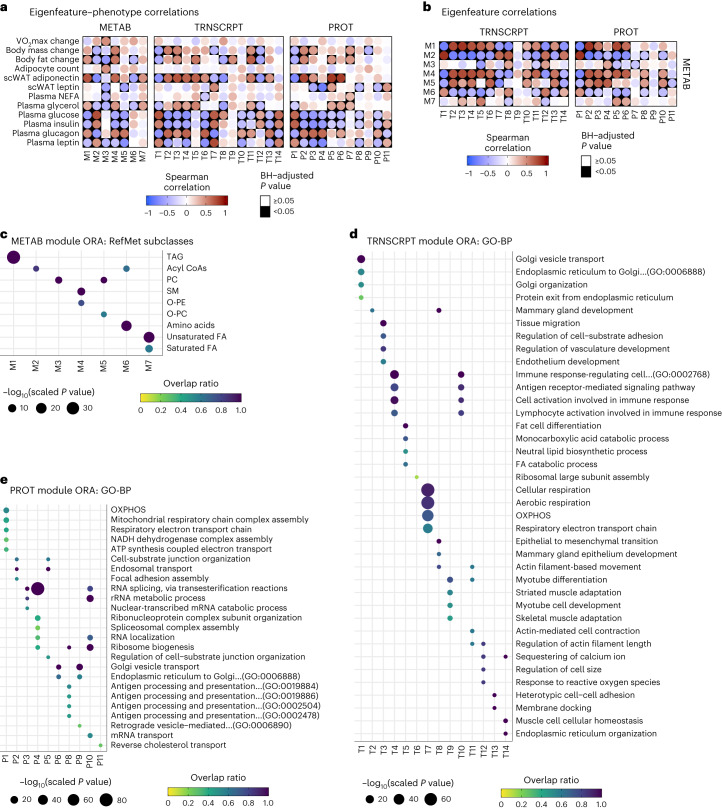
Integrative phenotypic–omic responses to ExT. **a**, Heat maps of Spearman correlations between MEs and clinical measurements. Change in phenotypic measures represents post − pre differences. **b**, Heat maps of Spearman correlations between the metabolomics MEs and the MEs of the other -omes. In both **a** and **b**, statistical significance was determined by two-sided Student’s *t*-tests of the transformed correlations and *P* values were adjusted to control the FDR using the BH procedure. **c**, All over-represented RefMet chemical subclasses in each metabolomics WGCNA module. **d**,**e**, Top over-represented GO-BP terms in each transcriptomics (**d**) or proteomics (**e**) WGCNA module.

**Extended Data Fig. 6:**
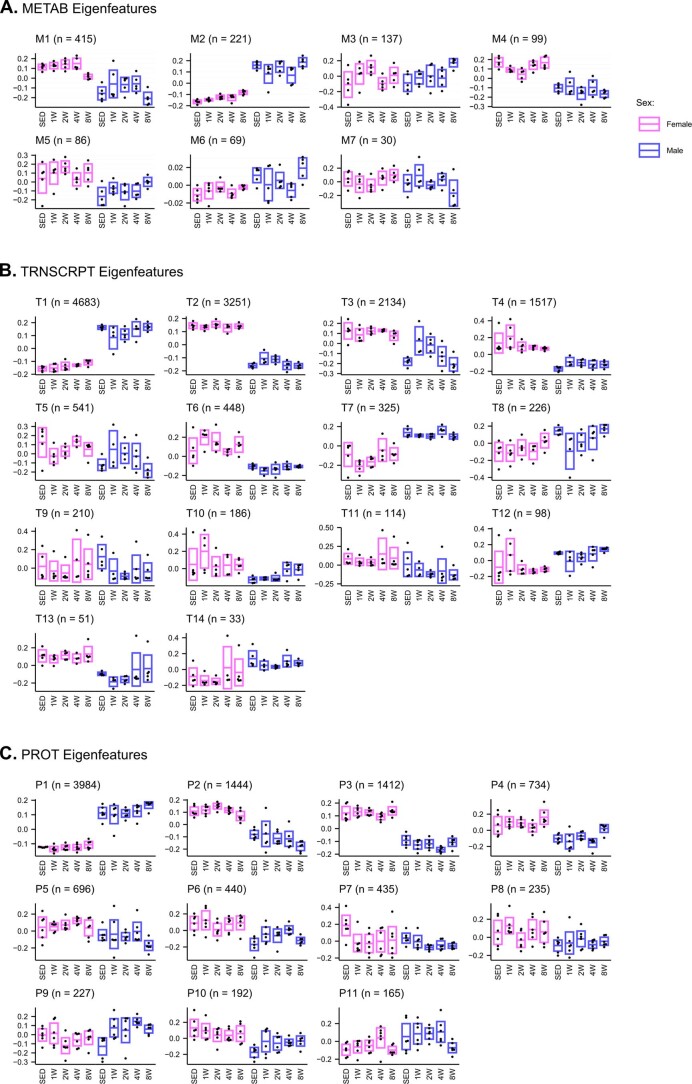
WGCNA module eigenfeatures. **a–c)** Plots of the module eigengenes (MEs) from the metabolomics (**a**), transcriptomics (**b**), and proteomics (**c**) WGCNA results. The size of each module is displayed along with the module labels. Boxes represent 95% confidence intervals for the mean of each group.

To integrate -omics datasets, we used WGCNA to generate modules (groups of highly correlated features) for metabolomics/lipidomics, proteomics and transcriptomics datasets (Extended Data Fig. 6a–c). We then assessed correlations of the module eigenfeatures (MEs), the principal eigenvector of each module, with select clinical plasma analytes and other phenotypic markers (Fig. 4a), as well as correlations between metabolomics and other -omics MEs (Fig. 4b). Afterwards, over-representation analysis (ORA) was performed to test for localization of GO terms or RefMet chemical subclasses in each module.

#### Metabolomics

The ME of the largest metabolomics/lipidomics module, M1, was higher in females throughout training (Extended Data Fig. 6a); M1 was over-represented by long-chain (>48 carbons) TAG species (Fig. 4c and Supplementary Table 8f). In addition to M1, other modules with MEs higher in females were M4 (over-represented by SM and O-PE species) and M5 (over-represented by PC and O-PC species) (Extended Data Fig. 6a). The M1, M4 and M5 module MEs correlated negatively with plasma glucose and the M1 and M4 MEs correlated positively with scWAT adiponectin and change in fat mass (Fig. 4a). This suggests a relationship between these modules with glucose regulation and lipid storage. The second largest module, M2, was over-represented by acyl-CoA species (Fig. 4c); its ME was higher in males and negatively correlated with scWAT adiponectin and changes in body fat (fat loss) (Fig. 4a). M3 contained predominantly 38–40 carbon PC species (Fig. 4c) and its ME increased at 8 weeks in males (Extended Data Fig. 6a). The MEs of M3 and the amino-acid-containing M6 module correlated positively with scWAT adipocyte count per field (reduced adipocyte size) and negatively with body fat changes (Fig. 4a,c), suggesting correlation with adipocyte lipid mobilization and utilization.

#### Transcriptomics

T1 was the largest transcriptomics module and its ME was highest in males and slightly increased with training in females (Extended Data Fig. 6b and Supplementary Table 8a). T1 was over-represented by terms relating to vesicle export and recycling processes, including autophagosome and protease terms (Fig. 4d and Supplementary Table 8d). T1 also contained terms relating to mitochondrial complex assembly and FA catabolic processes; its ME negatively correlated with the ME of the TAG-localizing M1 metabolomics module (Fig. 4b and Supplementary Table 8c,f), indicative of an inverse relationship between the aforementioned processes and scWAT TAG storage. The T7 ME was also higher in males and the module was over-represented by aerobic respiration terms (Fig. 4d and Extended Data Fig. 6b). This ME was negatively correlated with the TAG-localizing M1 module and positively correlated with the ME of the acyl-CoA-containing M2 ME (Fig. 4b and Extended Data Fig. 6b). The MEs of T2–T4 were higher in females and correlated with scWAT adiponectin protein abundance (Fig. 4a and Extended Data Fig. 6b). T2 and T3 were over-represented by terms related to developmental processes (Fig. 4d), including markers of adipogenic progenitors (*Cd34*, *Dpp4*, *Icam1* and *Pdgfrb*), suggestive of elevated adipogenic potential or different preadipocyte phenotypes in female scWAT. The T5 ME also correlated with the ME of the TAG-localizing M1 and remained relatively stable in females, increasing and then decreasing at 8 weeks in males, which resulted in elevated ME abundance in females relative to males at 8 weeks (Fig. 4b and Extended Data Fig. 8b). ORA of T5 revealed localization of transcripts involved in lipid storage and fat cell differentiation (*Fabp4*, *Lipe*, *Cebpa*, *Plin1*, *Pparg*, *Adipoq*, *Lpl* and *Slc2a4* (*Glut4*)). T4 and T10 were over-represented by immune signaling terms (most notably, lymphocyte activation) that increased only in males with prolonged training (T4 transcripts, *Btk*, *Cd28*, *Cd8a*, *Ctla4*, *Klrd1*, *Klrk1* and *Rela* (*Nfkb*); and T10 transcripts, *Ptprc* (*Cd45*), *Cd19*, *Cd22*, *Foxp3*, *Il21*, *RT1-DOa* and *Themis*) (Fig. 4d and Extended Data Fig. 6b). Notably, both MEs were negatively correlated with scWAT and plasma leptin levels (Fig. 4a), potentially suggestive of lymphocyte-associated lipolysis.

#### Proteomics

The ME of the largest proteomics module, P1, was much higher in males and increased in both sexes with training (Extended Data Fig. 6c and Supplementary Table 8b). P1 was dominated by terms relating to mitochondrial aerobic respiration/OXPHOS (Fig. 4e and Supplementary Table 8e). The P1 ME was negatively correlated with fat and body mass, the TAG-containing M1 ME and scWAT adiponectin levels (Fig. 4a,b and Supplementary Table 8c,f), again illustrating a negative association between lipid oxidation and scWAT TAG abundance. Notably, P1 also contained proteins related to peroxisomal tethering and biogenesis (Pex11a, Pex13 and Pex19), proteasomal proteins and activation of the innate immune system (for example, azurophil granule), suggestive of an effect of immune signaling to promote α and β oxidation in scWAT with ExT. The MEs of the next largest modules, P2–P4 and P6, were more abundant in females; their MEs correlated with changes in body fat and scWAT adiponectin levels (P2–P3) and were negatively correlated with plasma glucose, insulin and leptin (P2–P4) (Fig. 4a). The P6 module contained proteins related to lipid synthesis (Gpat3, Dgat2, Acsl1, Elovl1, Agpat2–4 and Mboat7) and insulin-regulated glucose transporter GLUT4 (Slc2a4), which were correlated positively with the TAG-containing M1 ME and negatively with the amino acid and acyl-CoA-containing M6 module ME. Together, our analyses reveal molecular hubs that mediate a sexually dimorphic response to ExT in the scWAT.

### Lipid-regulatory networks display sexual dimorphism with ExT

**Fig. 5:**
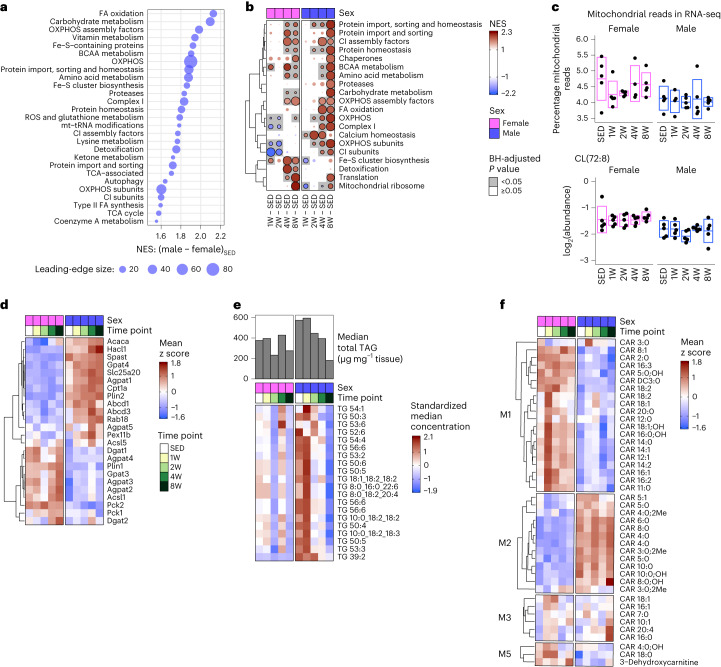
Integrative -omics reveals sexual dimorphism in scWAT mitochondrial metabolism and lipid recycling with ExT. **a**, Top significantly enriched (FDR < 0.05) MitoCarta terms from the proteomics sedentary male versus sedentary female FGSEA results. Points are scaled according to the number of genes in the leading-edge subset (the set of genes from each term that contributed to the enrichment score). Terms were only significantly enriched in males relative to females, so all points are colored blue. **b**, Heat map of the top MitoCarta terms that were most significantly enriched in any of the eight trained versus sedentary control comparisons from the proteomics FGSEA results. **c**, Percentage of reads from mitochondrial genes calculated from the raw transcriptomics data before any filtering (top) and the log_2_-transformed sample-level values of cardiolipin CL(72:8) (bottom). Both panels display 5 (or 4, following outlier removal as described in the ‘Differential analysis’ section in the Methods) biologically independent samples (rats) per group with boxes representing the mean ± s.d. **d**, Proteins involved in lipid metabolism. Values shown are the group means of the standardized sample-level protein values. **e**, Bar plot of the median total TAG concentration (μg mg^−1^ tissue) in scWAT samples of male and female rats at each time point. The heat map displays the standardized median concentration (peak area normalized by internal standard) of the top 20 most-abundant TAG species. **f**, Heat map of acylcarnitine species grouped according to their metabolomics WGCNA module. Values shown are the group means of the standardized sample-level values for each species.

**Extended Data Fig. 7:**
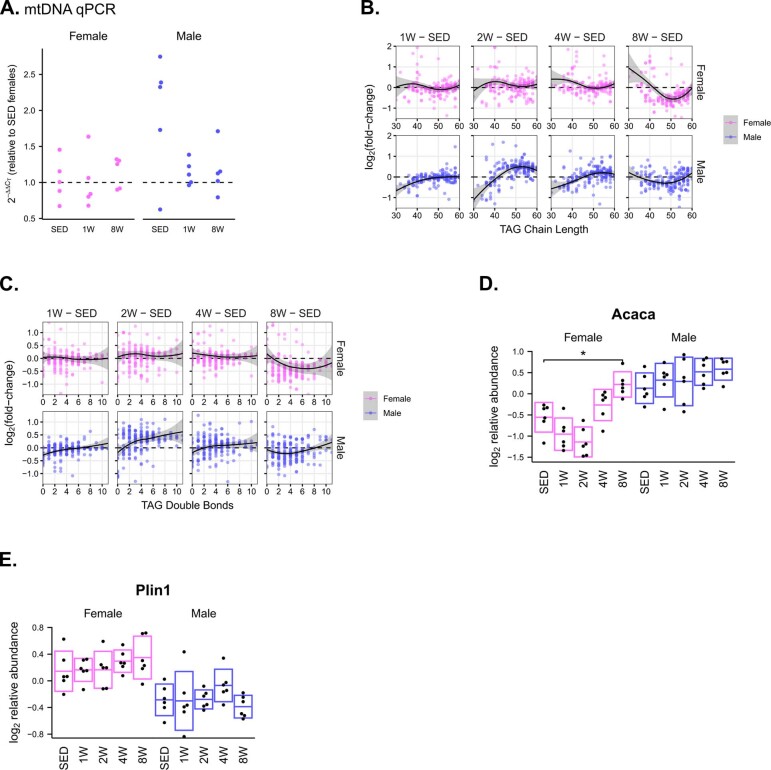
Sexual dimorphism in lipid dynamics with ExT. **a)** mtDNA 2^−ΔΔ*C*T^ values as measured by real-time qPCR. **b–c)** Changes in chain length (**b**) and double bond content (**c**) of TAG species in male and female rats after 1, 2, 4, and 8 weeks of exercise training. Loess curves are included with 95% confidence bands. The y-axes have been restricted to show patterns more clearly, so not all points are visible. **d–e)** log_2_ relative abundances of Acaca and Plin1 proteins (n = 6 per group). Boxes are 95% confidence intervals for the means, and an asterisk indicates a significant (BH-adjusted p-value < 0.05) difference between group means from the proteomics differential analysis results.

FGSEA of the MitoCarta3.0 database^36^ provided deeper insight into the impact of sex on mitochondrial metabolism in scWAT. Consistent with our previous FGSEA findings, SED males showed strong enrichment of MitoCarta terms compared to SED females (Figs. 2c and 5a and Supplementary Table 3c), including increased abundance of proteins involved in FA oxidation, and carbohydrate and BCAA metabolism. With training, males showed robust enrichment of catabolic pathways such as BCAA and carbohydrate metabolism and FA oxidation (Fig. 5b and Supplementary Table 5c). In contrast, females displayed greater enrichment of mitochondrial ribosome and translation pathways with ExT, displaying unique increases in Fe–S cluster biosynthesis and detoxification terms (Fig. 5b). Despite changes in mitochondrial metabolism pathways in both sexes, mitochondrial content as assessed by mitochondrial DNA content, percentage of RNA sequencing (RNA-seq) reads mapping to mitochondrial genes and cardiolipin CL(72:8) abundance were unaffected by training in either sex (Fig. 5c and Extended Data Fig. 7a). This suggests that mitochondrial abundance in scWAT was not altered by ExT in either sex.

Specific analysis of proteins regulating scWAT lipid recycling (re-esterification and lipolysis) revealed sexual dimorphism in long-chain acyl-CoA synthetase (Acsl) isoforms, an enzyme family that regulates metabolic partitioning of fatty acyl-CoAs (Supplementary Tables 2b and 4b). Females expressed higher levels of the major WAT isoform Acsl1 than males (Fig. 5d). Abundance of glycerol-3-phosphate acyltransferase (Gpat) isoforms (the rate-limiting enzymes of glycerolipid synthesis) also displayed sexual dimorphism, with Gpat3 higher in females compared to males at all time points and Gpat4 higher in males (Fig. 5d). Gpat3 is responsible for ~80% of the enzyme activity in WAT and is expressed during adipogenesis^37^; conversely, the male-dominant isoform Gpat4 is predominant in brown adipose tissue (BAT)^37^. Agpat, an enzyme family involved in glycerolipid and phospholipid synthesis, also displayed sexual dimorphism, with Agpat1 and Agpat5 highest in males and Agpat2–Agpat4 highest in females. Increased levels of Agpat2 in females suggests a lipid-storing phenotype, as this is the most abundant Agpat isoform in WAT, whereas Agpat1 (predominant in males) has bi-directional enzymatic activity in lipid synthesis^38^. Diglyceride acyltransferases (Dgat) and phosphoenolpyruvate carboxykinase (Pck) are also key re-esterification enzymes. Consistent with fat mass preservation, females displayed higher levels of Dgat1 and Pck2 at all time points (Fig. 5d). The lipid-synthesis protein Acaca (Acc1) was elevated in SED male scWAT and increased with 8 weeks of training in females (Fig. 5d and Extended Data Fig. 7d).

These changes in lipid esterification enzymes were complemented by differences in the perilipin (Plin) protein family, regulators of LD surface stability and accessibility for lipolysis, as Plin1 was upregulated in females (Fig. 5d, Extended Data Fig. 7e and Supplementary Tables 2b and 4b) and Plin2 in males (Fig. 5d). Plin1 is predominant on the surface of LD of large adipocytes and, in its inactive state, limits LD accessibility and lipolysis. Plin3, with roles in limiting lipolysis^39^, was also elevated in female scWAT. Conversely, the male-dominant Plin2 displays greater permissiveness toward lipolysis^39^. Together, this suggests that male scWAT is enriched in proteins favoring hydrolysis of lipid droplets, whereas female LD protein isoforms are indicative of a storage phenotype. Further emphasizing the catabolic effects of ExT on the male scWAT proteome, males displayed enrichment in key lipid oxidation proteins, including those involved in β-oxidation (Cpt1a, Slc25a20, Acads and Acadvl), lipid peroxisomal α- and β-oxidation (Abcd3 and Hacl1), LD tethering to peroxisomes (Spast and Abcd1) and peroxisome biogenesis (Pex11b) (Fig. 5d).

Finally, we examined changes in scWAT TAG abundance and other metabolomic markers of lipid metabolism. Consistent with decreased fat mass, plasma leptin and adipocyte size distribution, males decreased median total scWAT TAG abundance by ~70% after 8 weeks of ExT (Fig. 5e; bar plots). While females did not show substantial changes in scWAT TAG levels over the same time course (Fig. 5e), they exhibited changes in TAG composition. This is indicative of scWAT TAG remodeling or recycling with ExT in the absence of reduced fat mass (Fig. 5e and Extended Data Fig. 7b,c). This was characterized by an increase in short-chain (SC) and MC TAG abundance in 4- and 8-week-trained females, with decreases in long-chain (LC) TAG species (>40 carbons) at 8 weeks. Conversely, males displayed a reduction in SC TAGs in the early training response, indicated by a reduction in 30–40 carbon species (Extended Data Fig. 7b,c). At 8 weeks of ExT in males, the abundance of SC species was not different from SED and LC TAG species decreased, albeit to a lesser extent than in females (Fig. 5f and Extended Data Fig. 7b,c). Acyl-CoA and acylcarnitine species measured in this study report on the metabolism of FAs (Fig. 5f and Extended Data Fig. 4a). Females had higher levels of nearly all species of MC and LC (>8 carbons) acylcarnitines at all training time points, whereas males had higher levels of SC acylcarnitines, representing products of complete FA oxidation, with the exception of C2 (acetyl) (Fig. 5f). Females also displayed a robust increase in MC and LC acylcarnitines at 1 week, suggestive of reduced FA oxidation, consistent with reduced levels of OXPHOS proteins. These profiles are consistent with activation of FA oxidation to consume LC acylcarnitines and produce SC acylcarnitines in males and preservation of LC species in females, possibly for recycling to TAGs for storage. Together, changes in scWAT lipid metabolites are indicative of lipid utilization and depletion of fat stores in males versus lipid recycling to preserve fat mass in females.

## Discussion

The molecular hubs that differentiate male and female WAT and its response to ExT, remain largely unexplored. Building upon findings from the recently published MoTrPAC study^13^, here we use a multi-omic approach to comprehensively analyze the effects of sex and progressive endurance training on the biology of scWAT. Among 18 solid tissues with extensive -omic profiling in the same rats, the notable sexual dimorphism in the scWAT response to ExT described here contrasts with multi-omic responses in other tissues, such as the striated muscle, which displayed more consistent responses across sexes^13^. This led us to perform a scWAT-tissue-focused analysis to gain deeper insight into molecular hubs driving sexual dimorphism at rest and in response to progressive ExT. We show that male rat scWAT displays enrichment in markers of aerobic metabolism and lipid utilization in the sedentary state that are further increased with training, likely contributing to enhanced depletion of fat stores in males relative to females following 8 weeks of ExT. In contrast, the scWAT of females displays enrichment in markers of adipogenesis, insulin signaling and developmental and transcriptional/translational regulatory processes in sedentary and trained states, which likely impacts differential phenotypic responses to ExT. Utilization of WGCNA highlights molecular modules correlated with sexually distinct phenotypic responses, including changes in key regulatory hormones, metabolites and adipokines indicative ofdynamic molecular modules linked to lipid sparing and lipid utilization. We also identify differences in lipid-regulatory protein isoforms across sexes, with male scWAT having an increased abundance of BAT-associated protein isoforms in sedentary and trained states, despite no overt browning response. Together, our findings describe sexual dimorphism in the scWAT molecular landscape and the progressive response to ExT, thereby offering a unique resource for translational insight into factors linking scWAT biology to sex-stratified disease risk.

Despite increased fat mass, females have a reduced risk of cardiometabolic disease relative to age-matched male counterparts, in part attributed to the cardioprotective role of their increased scWAT relative to visceral WAT mass^9,10,12,18^. Increased scWAT adipogenesis and lipid deposition in females is associated with reduced inflammation and improved glucose homeostasis and lipid profiles^10,16^. Our study offers the opportunity to identify molecular and cellular regulators driving lipid buffering and insulin sensitivity in females versus males, with potential relevance in explaining mechanisms of protection against metabolic-related diseases in women before menopause. Despite the more robust catabolic response to training in males, females display characteristics of healthier scWAT, such as molecular features indicative of increased adipogenesis, lipid buffering and recycling capacity and insulin signaling^9,10,12^. Utilizing WGCNA, we substantiate this model by showing associations between lipid storage, glucose uptake and adipogenic molecular modules in females, with increased scWAT TAG content and reduced levels of plasma glucose. Given these metabolically advantageous phenotypes in female scWAT, increased lipid catabolism in male scWAT may be critical to promote metabolically favorable training adaptations.

Although WAT is one of the most sexually dimorphic tissues^13,14^, few studies have examined differential adaptability of scWAT to endurance exercise between sexes employing an integrative, multi-omic strategy. Another aspect of this research includes studying the progressive scWAT ExT response in adult rats, as training adaptations in young mice of a single sex and training time point are traditionally studied. Addressing this gap, our work identifies marked sex-specific differences in temporal transcriptional, proteomic and phosphoproteomic signatures in the scWAT in response to ExT. For example, at the transcriptional level, males displayed the largest response at 2 weeks of ExT (529 differential transcripts), whereas in females this occurred at 8 weeks (193 differential transcripts). Moreover, when compared to females, males had ~66% more differentially expressed transcripts throughout training (788 versus 474). At the proteomic level, males had ~2.5 times more proteins that were impacted by training compared to females (1,454 versus 574), with the proteomic response peaking at 2 weeks of ExT in females (221 features) and progressively increasing at 4 and 8 weeks in trained males (571 and 654 features, respectively). The temporal metabolomic response to training was the most consistent between the sexes, with both males and females displaying the greatest number of differential features at 8 weeks (females, 139 and males, 211) and 2 weeks (females, 135 and males, 161). Our findings are consistent with previous literature demonstrating a strong ExT-mediated -omic response in scWAT^7,17,40^.

Notably, this work provides a multi-omic view of GO terms, facilitating broad insight into molecular regulators of the scWAT progressive training response. FGSEA revealed early (1 week) enrichment of transcriptional and translational regulatory terms in females, whereas the 1-week response in males was enriched for GO terms related to tissue remodeling, including transcripts indicative of angiogenesis. Both sexes shared early increases in PC and O-PC species and terms related to adaptive immune cell activation. ORA of WGCNA modules gave results similar to those from FGSEA, indicative of an early adaptive immune response in females and males (T4 ME), which correlated with MEs of the plasmalogen-containing M4 and M5 modules. Plasmalogens are a major phospholipid class in cellular membranes, affecting cellular structural integrity, lipid raft formation and protein scaffolding and thus likely play an important role in transducing cellular signals and adipose remodeling. In lean adipose tissue, immune cells have recently been found to have positive tissue regulatory roles on adipocyte metabolism, lipid utilization and flux^6,41,42^, where depletion of regulatory T cell subsets contribute to insulin resistance^42^. Similarly, we observed an inverse association between plasma glucose and regulatory T cell term-containing T10 ME. Therefore, examining the role of plasmalogens as markers and/or regulators of immune cell subsets in the scWAT microenvironment is ripe for investigation.

The impact of sex on adipose tissue metabolism is likely multifactorial. While sex hormones clearly exert an effect on adipogenesis, lipolysis and re-esterification^9,10,18^, other factors such as genetic imprinting and embryonically established tissue-resident cellular populations likely contribute to sex differences in systemic metabolism^9,43,44^. We observed substantial sexual dimorphism in the scWAT of sedentary rats, where females displayed enrichment in transcriptomic terms relating to developmental processes and immune cell phenotypes, suggesting that the cellular landscape of the scWAT is intrinsically sexually dimorphic. In the genetically diverse hybrid mouse diversity panel cohort, ex vivo WAT mitochondrial function differs based on genetic background in a sex-dependent manner^44^. This emphasizes that gene by sex hormone interactions imprint metabolic phenotypes potentially through establishment of distinct cellular phenotypes. Recently, WAT has been shown to have substantial heterogeneity of adipocyte, preadipocyte and various immune cell populations that change with obesity and high-fat feeding^3^. Future studies performing cellular deconvolution or single cell analyses using this or other datasets may help to define how sex and training impact scWAT cellular populations and their relation to systemic metabolism. Fluctuations in estrous cycle hormones also impact training-associated phenotypes, such as lipid utilization and recycling^9,18^. In our study, training and tissue collection were temporally staggered. It is thus expected that female rats are at different stages of their 4–5-d estrous cycle regardless of time point, such that no single group is biased to any stage of the cycle. While this may have contributed to higher variance for some measures in females, it also means that our findings represent the comprehensive impact of training on measures throughout the female estrous cycle.

Phosphoproteomics identified probable molecular transducers of the sexually dimorphic scWAT response to training. With ExT, male scWAT exhibited changes consistent with enhanced lipid-mobilizing AMPK activity^45^, whereas female scWAT exhibited profiles consistent with enrichment of anabolic AKT and mTOR activity. In females, overall enrichment of anabolic multi-omic signatures (for example, amino acids, insulin-permissive signaling molecules, adipogenic MEs and predicted AKT and mTOR kinase activity) illuminate metabolically favorable mechanisms by which scWAT adapts to training, even in the absence of fat loss, to promote cardiometabolic health and protect reproductive fitness. Supporting these observations, mTORC1 signaling promotes scWAT lipogenesis and adipogenesis^31^. Notably, ablation of mTOR and LKB prevents diet-induced obesity, yet promotes insulin resistance^46^. Further supporting metabolically beneficial adaptations in female scWAT with ExT was the consistent downregulation of the insulin signaling repressor, Grb14, at all training time points. Estradiol inhibits insulin-induced Grb14 expression^47^, highlighting a potential mechanism by which female sex hormones might contribute to increased scWAT insulin sensitivity. Females did display increased scWAT metabolism evident through MitoCarta analyses with OXPHOS proteins increasing in females with training, albeit less than that of males. Female scWAT displayed TAG species remodeling, likely indicative of lipid utilization and subsequent re-esterification, reflected by the increased abundance of short-to-medium chain TAGs. This is further supported by WGCNA, which localized transcripts related to neutral lipid biosynthetic processes to the T5 module, the ME of which was highly correlated with change in fat mass and the M1 LC TAG-localizing metabolomics ME.

Our work provides a critical foundation for understanding the coordinated molecular responses to chronic endurance exercise, as these rats were intentionally studied 48 h following the last bout of exercise to capture durable adaptations to ExT. Translation of these findings to outbred rodent and human models are key next steps for building upon this work. Our findings highlight that, while adaptations in scWAT in response to physical activity were largely dimorphic, both male and female rats display metabolically favorable responses to ExT. In the present study, we observed no significant decrease in fat mass in female rats in response to training, but scWAT was significantly remodeled to display a healthier phenotype and a lack of ExT led to an increase in fat mass in both sexes. We note that longer training duration or volume can promote fat loss in females and that such responses are likely dependent on genetic background and baseline fat mass^40,48^. Further, our study focused on a single subcutaneous fat depot in rodents; other fat depots may respond differently to chronic training^12,49,50^. Our study design did not control for or measure food intake or ambulatory movement, so we cannot determine whether the sexual dimorphism in the observed training response was affected by changes in caloric consumption and non-exercise energy expenditure. Finally, given the scope of the study, we did not have age-matched sedentary controls at all time points and chose to age-match only for the 8-week-trained group. We based this on our expectation that most training effects would occur at this later time point and that transcriptomic changes associated with aging only become evident at 12 months or older^51^. Nevertheless, future studies may benefit from inclusion of age-matched controls at all training time points.

In summary, our study characterizes sexually distinct temporal dynamics in the multi-omic response of scWAT to ExT. Our data represent a companion analysis of the larger MoTrPAC multi-omic study of training responses across multiple tissues^13^. The focused analysis of scWAT summarized here highlights the utility of the dataset to provide insight into tissue-specific adaptations, yielding knowledge to drive new hypothesis-based research. Our study identifies opportunities for future investigation of causal links between -omics clusters and phenotypic responses to training, including the impact of sex on such pathways. Identifying sex-specific and sex-conserved scWAT responses to training creates a framework for deeper understanding of adipose tissue biology and re-emphasizes the need to consider biological sex when strategizing precision-based interventions.

## Methods

### Animals

Adult male and nulliparous female Fischer 344 (F344) inbred rats were obtained from the National Institute on Aging (NIA) rodent colony. Rats of the same sex were housed two per cage (146.4 in^2^ of floor space) in ventilated racks (Thoren Maxi-Miser IVC Caging System) on Tekland 7093 Shredded Aspen bedding and fed the LabDiet 5L79 pelleted diet, which are the standard bedding and diet used at the NIA rodent colony. The animal housing room was monitored daily and maintained at 68–77 °F and 25–55% humidity. Rats were adapted to a reverse dark–light cycle with lights off at 9:00 and lights on at 21:00 for a minimum of 10 d, and rats were handled daily by staff to reduce stress and promote acclimation to human handling and the research facility. Red lights were used during the dark cycle to provide adequate lighting for routine housing tasks, rodent handling and training to ensure rats were handled during their nocturnally active phase. All animal procedures were approved by the Institutional Animal Care and Use Committee at the University of Iowa.

### Treadmill familiarization and exercise training

Before ExT, all animals underwent a 12-d treadmill familiarization consisting of 5–10 min of low-intensity (6–10 m min^−1^) daily exercise. Only rats able to maintain treadmill compliance (for example, continuous forward running) were randomized to control or intervention groups. Following treadmill familiarization, ExT (Panlab five-lane rat treadmill, Model LE8710RTS, Harvard Instruments) began at 6 months of age and lasted for 1, 2, 4 or 8 weeks. Training began and ended on a staggered schedule over a 3–5-d period to accommodate the termination schedule, so female rats were at different stages of their estrous cycle upon tissue collection, regardless of time point. Rats were trained for five consecutive days per week at 70–75% of VO_2_max using a progressive treadmill protocol that eventually reached a duration of 50 min d^−1^. Sedentary control rats were placed on the treadmills for 15 min d^−1^ at a speed of 0 m min^−1^ for five consecutive days per week, following a schedule similar to the 8-week-trained rats, to control for any non-exercise-related treadmill effects. Rats unable to exercise for at least 4 d per week were removed from the study.

### Body composition

Body composition was determined for all rats 13 d before the start of training using the minispec LF90II Body Composition Rat and Mice Analyzer (Bruker). Post-training body composition was determined for rats in the control, 4- and 8-week-trained groups 5 d before tissue collection.

### VO_2_max

VO_2_max was determined before the onset of training in all rats and during the last week of training for the 4- and 8-week exercise groups. Rats were acclimated to a single-lane enclosed treadmill (Columbus Instruments Metabolic Modular Treadmill) 2 d before testing. On the day of testing, the rat was placed in the treadmill, and testing began once oxygen consumption was stabilized. VO_2_, VCO_2_ and respiratory exchange ratio (RER) were recorded at 5-s intervals while in the treadmill and blood lactate levels were recorded at 2-min intervals. Testing began with a 15-min warmup at a speed of 9 m min^−1^ and 0° incline. Following this, the incline was increased to 10° and the treadmill speed was increased by 1.8 m min^−1^ every 2 min^19^. Post-training males and females remained at speeds of 23.4 and 27 m min^−1^, respectively, for a total of 4 min; the first 2 min were spent at an incline of 10° and the last 2 min at an incline of 15°, which was then maintained for the duration of the testing period. Criteria for reaching VO_2_max was a plateau in oxygen uptake despite increased workload, RER above 1.05 and a non-hemolyzed blood lactate concentration ≥6 mmol l^−1^ (ref. ^19^).

### Statistical analyses of phenotypic measures

Multiple linear regression models with sex, time point and their interaction were included as predictors of the post − pre differences for various phenotypic measures: VO_2_max relative to whole body mass (recorded on the day of the VO_2_max test) or NMR lean mass, body mass (recorded on the same day as NMR body composition measures), NMR fat mass and fat percentage. Inverse group variances were included as weights in the models if heteroscedasticity was observed, and model parsimony was achieved by examining *F*-tests and model diagnostic plots (such as residuals versus fitted, scale–location and quantile–quantile). Then, we tested the null hypothesis that the mean post − pre difference of each of the SED, 4-week- and 8-week-trained groups were equal to 0 by sex. The Holm multiple comparison procedure was applied to each set of three comparisons by sex to control the family-wise error rate.

### Tissue collection

Tissues were collected from all rats at 48 h following the last training session. As training began and ended on a staggered schedule (see ‘Treadmill familiarization and exercise training’ section), collection was also performed over the course of a 3–5-d period for each experimental group. On the day of collection, food was removed at 8:30, 3 h before the the start of dissections, which occurred between 11:30 and 14:30. Rats were sedated with inhaled isoflurane (1–2%), during which blood was drawn via cardiac puncture and subcutaneous white fat (inguinal fat depot from right side) was removed and immediately frozen in liquid nitrogen, placed in cryovials and stored at −80 °C. Removal of the heart resulted in death.

### Measurement and statistical analyses of clinical analytes

We measured a set of nine common clinical analytes in plasma: glucose, lactate, glycerol, total ketones, NEFAs, glucagon, insulin, leptin and corticosterone. The first five were measured using a Beckman DxC 600 clinical analyzer with reagents from Beckman and Fujifilm Wako (total ketones and NEFAs). The others were measured in immunoassays using commercial kits from Meso Scale Discovery and Alpco (corticosterone). For each of the analytes, we first examined their mean–variance relationship. Informed by these results, we fit log-link gamma (glucagon only) or Gaussian generalized linear models (GLMs). For the Gaussian GLMs, we included reciprocal group variances as weights to account for heteroscedasticity. Regression diagnostic plots (such as residuals versus fitted, scale–location and quantile–quantile) were checked to ensure all assumptions were met. Then, we compared each of the trained time points to their sex-matched sedentary controls using the Dunnett multiple comparison procedure if time point was included as a predictor. We also compared males to females by time point, applying the Holm multiple comparison procedure to each set of five *P* values. As a log link was used, results are presented as ratios of group means. Tests were only performed on the subset of samples selected for multi-omics analyses (*n* = 6 per experimental group) for glucagon, glucose, glycerol, insulin, leptin and NEFAs. The other analytes were not tested. Clinical analyte measures and results of statistical analyses are provided in Supplementary Table 1c–e.

### WAT histology

scWAT tissue samples were collected from the contralateral side of rats used for multi-omics analyses, embedded in OCT and flash frozen for histological analyses. Tissue samples were sectioned with a thickness of 15 μm in a cryostat, stained with hematoxylin and eosin and visualized with bright-field microscopy. A total of ten fields of view were imaged per sample using automated CellProfiler software^52^ to quantify adipocyte cell size. Adipocytes were binned in 5-μm intervals according to their diameter. A log-link negative binomial regression model that included sex, time point, diameter bin (ordered factor, ten levels) and their interactions as predictors was used to model the expected rate of adipocytes (offset of log(total adipocytes per sex and time point group)). The regression assumptions were checked with diagnostic plots (such as residuals versus fitted, scale–location and quantile–quantile). Within each bin, we compared the 4- and 8-week-trained time points to their sex-matched sedentary controls using the Dunnett multiple comparison procedure. The ratios of the expected values are shown in Extended Data Fig. 1d if the adjusted *P* values were below 0.05. Measures of adipocyte diameter, area and volume, as well as results of the statistical analysis are provided in Supplementary Table 1a,b.

### Multi-omic data generation and processing

Full details of the methods used for sample processing, data collection, data processing, data normalization and batch correction for transcriptomics, proteomics, phosphoproteomics and metabolomics/lipidomics platforms are described elsewhere^13^ and summarized in the Supplementary Methods, which also contain additional details for the statistical analyses described below.

### Differential analysis

**Extended Data Fig. 8:**
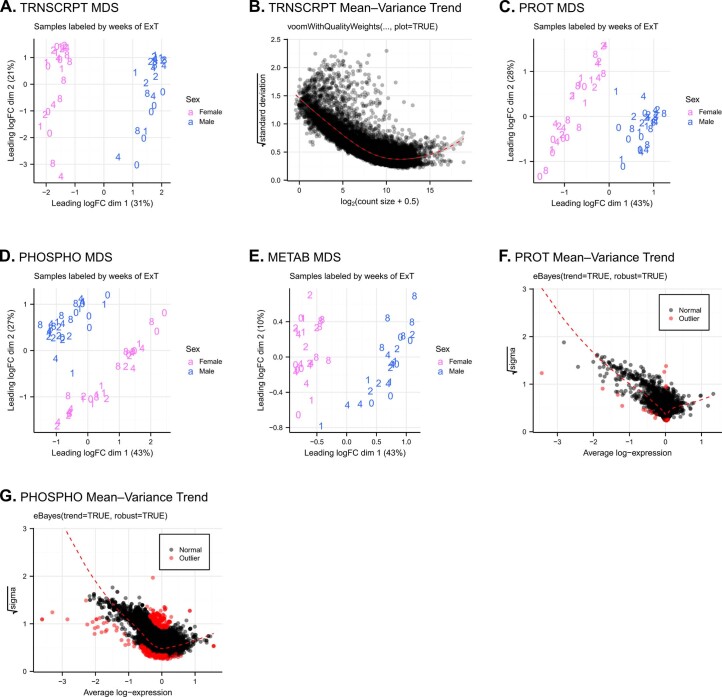
Differential analysis model indicators. **a)** Multidimensional scaling (MDS) plots where distances between points approximate the typical log_2_ fold-changes between transcriptomics samples. **b)** Scatterplot of the average log_2_ counts per million vs. the square-root of the residual standard deviations from *limma::voom*. The loess curve (dashed red line) approximates the mean–variance relationship. **c–e)** MDS plots where distances between points approximate the typical log_2_ fold-changes between proteomics (**c**), phosphoproteomics (**d**), or metabolomics (**e**) samples. **f–g)** Average log_2_ relative abundance vs. the square-root of the residual standard deviations of proteins (**f**) or phosphosites (**g**). The dashed red line indicates the robust mean–variance trend from the *limma::eBayes* step. This is the *ggplot2* equivalent of the *limma::plotSA* output.

RNA-seq data were analyzed using the edgeR^53^ and limma^54^ Bioconductor/R packages, following the workflow described by Law et al.^55^. Briefly, low-abundance transcripts were removed from the raw count data with edgeR::filterByExpr to estimate the mean–variance relationship more accurately. Then, a multidimensional scaling (MDS) plot was generated from the log_2_ TMM-normalized counts per million reads to explore the average log_2_ fold change between samples (Extended Data Fig. 8a). Two samples (90423017005, from a 1-week-trained male and 90410017005, from a 4-week-trained female) were identified as outliers in the principal-component analysis plots and MDS plots and removed^13^. Furthermore, higher variability was observed in females relative to males, and biological replicates (for example, all 4-week-trained males) appeared to cluster poorly. To account for this, limma::voomWithQualityWeights was chosen to simultaneously combine observation-level weights (predicted from the mean–variance trend in Extended Data Fig. 8b) with sample-specific quality weights. These weights are incorporated into the linear model to down-weight low-abundance observations and all observations from more variable samples, increasing power to detect differences^56,57^.

Proteomics, phosphoproteomics and metabolomics data were also analyzed using limma and differences in sample variability were again apparent from their respective MDS plots (Extended Data Fig. 8c–e). As such, sample-specific quality weights were calculated with limma::arrayWeights (method= ‘genebygene’) and incorporated into the linear models^58^. Additionally, RNA integrity number, median 5′-3′ bias, percent of reads mapping to globin and percent of PCR duplicates as quantified with unique molecular identifiers were included as covariates in the RNA-seq model after they had been mean-imputed and standardized^13^. For each -ome, we set up a no-intercept model containing the experimental group (each combination of sex and time point; factor with ten levels) and any covariates.

Following linear modeling with limma::lmFit, contrasts were constructed with limma::contrasts.fit to test sex-specific training responses (for example, 1-week-trained versus SED males; Supplementary Table 4a–d), baseline sexual dimorphism (SED males versus SED females; Supplementary Table 2a–d) and sexually dimorphic training responses (the sex by training interaction effect; Supplementary Table 6a–d). Then, robust empirical Bayes moderation was carried out with limma*::*eBayes to squeeze the residual variances toward a common value (RNA-seq) or a global trend (proteomics and phosphoproteomics; Extended Data Fig. 8f,g)^59^. For metabolomics, the empirical Bayes moderation was performed separately for each of the 13 platforms with robust = trend = TRUE (both FALSE if a platform measured fewer than ten metabolites).

To control the FDR, *P* values were adjusted across sets of related comparisons by -ome using the BH procedure. Counts of differential features (FDR < 0.05) from the timewise comparisons are shown in the UpSet plots (Fig. 3a,b).

### Fast gene set enrichment analysis

Gene set enrichment analysis (GSEA) is a rank-based approach that determines whether any a priori defined gene sets, such as genes involved in the same biological process or participating in the same pathway, display concordant changes in their expression, even when those changes are fairly modest^60^. FGSEA^21^ is a fast implementation of pre-ranked/gene permutation GSEA. It requires ranking metric values (the gene-level statistics) and a collection of gene sets. We chose the signed −log_10_-transformed *P* value (calculated from the differential analysis results for each contrast) as the ranking metric, where the sign indicates the direction of the log_2_ fold change.

For each contrast, ranking metric values were calculated at the level of individual features (proteins, transcripts and metabolites). Proteomic and transcriptomic-ranking metrics were aggregated to the Entrez gene ID level by taking the arithmetic mean. Any features that did not map to an Entrez gene were discarded before analysis.

For proteomics and transcriptomics, gene sets from the three C5:GO subcollections (BP, MF and CC)^61,62^ of the Molecular Signatures Database^63^ (MSigDB v.7.5.1) were considered for testing. For proteomics only, we additionally tested gene sets from the MitoCarta3.0 database^36^ (file ‘Human.MitoCarta3.0.xls’) (Fig. 5c,d). Human gene symbols were remapped to their rat orthologs (Entrez gene IDs) before analysis. Finally, the FGSEA procedure was applied to the metabolomics data by grouping metabolites according to RefMet chemical subclasses (for example, acylcarnitines and triacylglycerols), provided by the Metabolomics Workbench RefMet database (https://www.metabolomicsworkbench.org)^22^.

Results are provided in Supplementary Tables 3a–e, 5a–e and 7a–d.

### Kinase–substrate enrichment analysis

To infer kinase activity from our phosphoproteomics data, we utilized the curated kinase–substrate information provided by PSP^30^ (v.6.6.0.4). As data are markedly more comprehensive for humans than for rats, we first remapped the 30,304 quantified phosphorylation sites on rat proteins to human orthologs using the data generated by the MoTrPAC Study Group^13^ (Methods ‘Mapping PTMs from rat to human proteins’). This resulted in 19,137 unique phosphorylation sites on human proteins. FGSEA^21^ was then performed for each of the phosphoproteomics contrasts using the single-site-level signed −log_10_-transformed *P* value as the ranking metric. Kinase sets were constructed from the PSP kinase–substrate dataset by grouping sites phosphorylated by the same kinase. Only those kinases with at least three annotated substrate sites (excluding instances of autophosphorylation) were tested for enrichment. Results are provided in Supplementary Tables 3d and 5d.

### Weighted gene coexpression network analysis

WGCNA^64^ was carried out to identify non-overlapping groups of related proteins, metabolites and transcripts (hereby referred to as ‘modules’) (Supplementary Table 8a–c). The modules are arranged in descending order by size and labeled with the first letter of their respective -ome (M, T or P). MEs, the principal eigenvector of each module, were extracted and Spearman correlations were calculated between metabolomics/lipidomics and proteomics MEs, between metabolomics/lipidomics and transcriptomics MEs (Fig. 4b) and between each ME and select sample measures (Fig. 4a). We assessed the significance of each correlation by applying two-sided Student’s *t*-tests to the transformed correlations (Supplementary Methods) and adjusted these *P* values within each correlation matrix using the BH procedure.

### WGCNA module over-representation analysis

To characterize the WGCNA modules, ORA was performed in R with fgsea*::*fora^21^ on the same feature sets that were used for FGSEA. All Entrez or RefMet IDs from the appropriate WGCNA results (excluding those from the ‘gray’ modules) were set as the background for these hypergeometric tests. The top over-represented (scaled *P* value < 0.05) feature sets by module are shown in Fig. 4c–e. Full results are provided in Supplementary Table 8d–f.

### Mitochondrial DNA quantification

Quantification of mtDNA was performed and described by Amar et al.^65^. Briefly, real-time qPCR was performed in duplicate for each of the scWAT samples selected for -omics analysis. The ${2}^{-\Delta \Delta {C}_{T}}$ method^66^ was then applied to estimate the relative expression of the mitochondrial D-loop. As both target (D-loop) and internal control (β-actin) were amplified in the same well, Δ*C*_T_ was calculated as the mean of (*C*_T,D-loop_ − *C*_T,β-actin_) for each sample. Then, ΔΔ*C*_T_ values were obtained by subtracting each Δ*C*_T_ value by the mean Δ*C*_T_ of the SED female group (the calibrator). Kruskal–Wallis tests were performed on the ${2}^{-\Delta \Delta {C}_{T}}$ values separately by sex.

### Sample size and assumptions of statistical analyses

Experimental groups consisted of *n* = 5 biological replicates for metabolomics/lipidomics, transcriptomics and mtDNA data and *n* = 6 biological replicates for proteomics/phosphoproteomics, histology, plasma clinical analytes and phenotypic measures data. No statistical methods were used to predetermine sample sizes, but these sample sizes are consistent with what has been reported in previous rodent studies^3,7,18,46,67^.

For all statistical analyses, we first ensured that the relevant model assumptions were satisfied before proceeding with hypothesis testing. This is detailed in the vignettes of the MotrpacRatTraining6moWATData R package (Data Availability).

### Reporting summary

Further information on research design is available in the Nature Portfolio Reporting Summary linked to this article.

### Supplementary information

Supplementary Information Additional methods information related to data generation, processing and statistical analyses.

Reporting Summary

Supplementary Data 1 Data and statistics for WAT histology and plasma clinical analyte measures; and complete analysis results (DEA, FGSEA, WGCNA and ORA) for manuscript figures.

### Source data

Source Data Fig. 1 Unprocessed microscopy image for Fig. 1d.

## Figures and Tables

**Extended Data Table 1:**
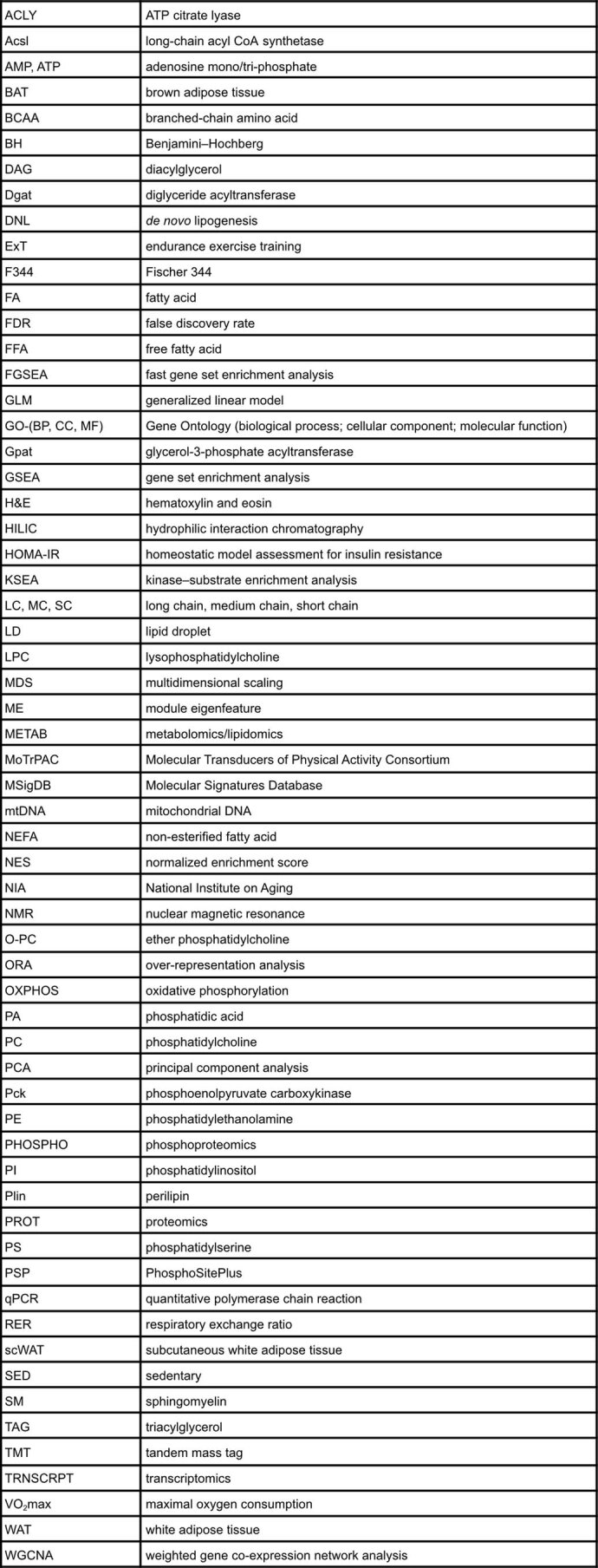
List of abbreviations and their meanings

## Data Availability

Processed data and analysis results are available in the MotrpacRatTraining6moWATData R package (github.com/MoTrPAC/MotrpacRatTraining6moWATData). MoTrPAC data are publicly available via motrpac-data.org/data-access. Data access inquiries should be sent to motrpac-helpdesk@lists.stanford.edu. Additional resources can be found at motrpac.org and motrpac-data.org. We used external datasets from MitoCarta3.0 (ref. ^36^) (Human.MitoCarta3.0.xls, accessed from https://www.broadinstitute.org/mitocarta/mitocarta30-inventory-mammalian-mitochondrial-proteins-and-pathways), MSigDB^63^ (v.7.5.1), PhosphositePlus^30^ (v.6.6.0.4; Kinase_Substrate_Dataset.xlsx, accessed 5 June 2022 from https://www.phosphosite.org/staticDownloads), the Metabolomics Workbench RefMet database^22^ (https://www.metabolomicsworkbench.org) and published data from The MoTrPAC Study Group^13^ and Amar et al.^65^. MoTrPAC data relevant to this manuscript is deposited in the following public repositories: **NCBI Gene Expression Omnibus: RNA-seq** Data types: processed pipeline outputs raw counts, merged peakset and coverage files. To review Gene Expression Omnibus accession code GSE242358, enter token abmhgaoktbmtjwv into the box. **Metabolomics Workbench: targeted and untargeted metabolomics** Data types: raw + results files Project ID PR001020 Project 10.21228/M8V97D http://dev.metabolomicsworkbench.org:22222/data/DRCCMetadata.php?Mode=Project&ProjectID=PR001020&Access=QhxG2479 **MassIVE: proteomics** Data types: raw + results files • MassIVE MSV000092911 (10.25345/C5R78603Q): MoTrPAC, Endurance Exercise Training Study in 6-Month-Old Rats (Global Protein Abundance in Gastrocnemius, White Adipose, Cortex, Lung and Kidney)• MassIVE MSV000092925 (10.25345/C52F7K23N): MoTrPAC, Endurance Exercise Training Study in 6-Month-Old Rats (Protein Phosphorylation in Gastrocnemius, White Adipose Tissue, Cortex, Lung and Kidney) Source data are provided with this paper.

## Code availability

R scripts detailing all processing and analysis steps are contained within the data-raw/ folder or in the vignettes and articles of the MotrpacRatTraining6moWATData R package (github.com/MoTrPAC/MotrpacRatTraining6moWATData). Helper functions used to perform analyses and visualize results are contained within the MotrpacRatTraining6moWAT R package (github.com/MoTrPAC/MotrpacRatTraining6moWAT). For a list of the core R/Bioconductor packages used, please consult the Software section of the Supplementary Methods.
