# Supplementary material for: Sexual dimorphism and the multi-omic response to exercise training in rat subcutaneous white adipose tissue
Source: Nat Metab. 2024 May 1;6(5):963–79. doi: 10.1038/s42255-023-00959-9 (PMC11132991; doi:10.1038/s42255-023-00959-9)
Supplement: Supplementary file 1 — Additional methods information related to data generation, processing and statistical analyses. [file 42255_2023_959_MOESM1_ESM.pdf]

nature metabolism

Resource

https://doi.org/10.1038/s42255-023-00959-9

Sexual dimorphism and the multi-omic response to exercise training in rat subcutaneous white adipose tissue

In the format provided by the authors and unedited

Supplementary Methods

Data generation and processing

For complete detailed descriptions of methods used for sample preparation and multi-omics data generation and processing at chemical analysis sites for this study, please see the associated MoTrPAC publication[1].

Randomization and Blinding
At all multi-omic analysis sites, an unblinded batching officer was responsible for randomization of the samples across batches of appropriate size for the analysis platforms in place. Randomized samples were blinded to all individuals involved in sample preparation, data generation, and initial data processing. Downstream quality control and data analysis were not performed blind to the conditions of the experiments.

RNA-sequencing
Total RNA was extracted from tissue lysates using a BiomekFx automation workstation. For blood samples, total RNA was extracted using the Agencourt RNAdvance blood specific kit (Beckman Coulter). RNA quantity and integrity was assessed with a Nanodrop (ThermoFisher Scientific, #ND-ONE-W), Qubit assay (ThermoFisher Scientific), and either Bioanalyzer or Fragment Analyzer. 500 ng of total RNA from each sample was used to generate libraries for RNA sequencing using the Universal Plus mRNA-Seq kit (NuGEN/Tecan #9133) and prepared with a Biomek i7 laboratory automation system (Beckman Coulter). Sequencing of pooled libraries was performed through 100bp paired-end sequencing using the Illumina NovaSeq 6000 platform (Illumina), targeting a sequencing depth of 35 millions read pairs per sample. Sequenced reads were demultiplexed with bcl2fastq2 (v2.20.0), adapters were trimmed with cutadapt (v1.18), pre-alignment QC metrics generated with FastQC (v0.11.8) and reads aligned using STAR (v2.7.0d). Quantification was performed using RSEM (v1.3.1).

LC-MS/MS proteomics and phosphoproteomics
Proteomics analyses were performed using clinical proteomics protocols described previously[2], with full details provided here[1]. scWAT samples were lysed and protein concentration was determined using BCA assay. Protein lysates were reduced with 5 mM dithiothreitol (DTT, Sigma-Aldrich) for 1 hour at 37 °C with shaking at 1000 rpm on a thermomixer, alkylated with iodoacetamide (IAA, Sigma-Aldrich) in the dark for 45 minutes at 25 °C with shaking at 1000 rpm, and diluted 1:4 with Tris-HCl, pH 8.0. Proteins were first digested with LysC endopeptidase (Wako Chemicals) at a 1:50 enzyme:substrate ratio (2 hours, 25 °C, 850 rpm), followed by digestion with trypsin (Promega) at a 1:10 enzyme:substrate ratio (14 hours, 25 °C, 850 rpm). Formic acid was added to a final concentration of 1% to quench digestion, after which peptides were desalted using Sep-Pac C18 columns (Waters) and BCA assay was used to determine final peptide concentrations.

Peptide aliquots (400 µg per sample) were resuspended to a final concentration of 5 µg/µL in 200 mM HEPES, pH 8.5 for isobaric labeling. Samples were randomized across the first 10 channels of tandem mass tag (TMT) 11-plexes (ThermoFisher Scientific), and the last channel (131C) of each multiplex was used for a common reference composed of a mix of peptides from all samples. TMT reagent was added to each sample at a 1:1 peptide:TMT ratio, and labeling proceeded for 1 hour at 25 °C with shaking at 400 rpm. After labeling QC checks, reactions were quenched with hydroxylamine and samples within each multiplex were combined and desalted with Sep-Pac C18 columns (Waters). Each combined TMT multiplex was then fractionated using high pH reversed phase separation and concatenated into 24 fractions. 5% of each fraction was removed for global proteome analysis, and the remaining 95% was further concatenated to 12 fractions for phosphopeptide enrichment using immobilized metal affinity chromatography (IMAC).

For mass spectrometry analysis of the global proteome, online separation was performed using a nanoAcquity M-Class UHPLC system (Waters) and a 25 cm x 75 µm i.d. picofrit column packed in-house with C18 silica (1.7 µm UPLC BEH particles, Waters Acquity). Samples were analyzed with a Q Exactive HF mass spectrometer (ThermoFisher Scientific). For phosphoproteome samples, online separation was performed with a Dionex Ultimate 3000 UHPLC system (ThermoFisher Scientific) and a 30 cm x 75 µm i.d. picofrit column packed in-house with C18 silica (1.7 µm UPLC BEH particles, Waters Acquity). Samples were analyzed with a Q-Exactive HFX mass spectrometer (ThermoFisher Scientific). Full information regarding elution gradients and instrument settings for global proteomics and phosphoproteomics samples is described elsewhere[1].

Log_2 TMT ratios to the universal reference were used as quantitative values for all proteomics features (full details of raw MS/MS data processing are described by the MoTrPAC Study Group[1]). Contaminant identifications and features not fully quantified in at least 2 TMT multiplexes were excluded from downstream analysis. Sample normalization was performed by median-centering and mean absolute deviation (MAD)-scaling within each sample, after which TMT multiplex batch effects were removed using the *limma::removeBatchEffect* function in R[3,4].

Untargeted metabolomics

Hydrophobic interaction liquid chromatography (HILIC) analyses of polar metabolites in the positive ionization mode were conducted at the Broad Institute of MIT and Harvard. 10 mg of cryopulverized tissue was homogenized in 300 µL of 10/67.4/22.4/0.018 v/v/v/v water/acetonitrile/methanol/formic acid containing stable isotope–labeled internal standards, and centrifuged at 9,000 x g for 10 min. Supernatants were injected onto a HILIC column (Waters) and analyzed using a Q-Exactive hybrid quadrupole Orbitrap mass spectrometer (ThermoFisher Scientific) operating in the positive mode. Raw data was processed for targeted peak integration using TraceFinder software (ThermoFisher Scientific) and with Progenesis QI software (Nonlinear Dynamics, Waters) for peak detection and integration of both metabolites with known identity and unknowns.

Reverse-phase and ion pairing profiling of polar metabolites was conducted at the University of Michigan. Non-pulverized tissue samples were weighed and homogenized in 1:1:1:1 methanol:acetonitrile:acetone:water (at a ratio of 1 mL per 50 mg tissue) using a sonicator. Samples were incubated on ice for 10 minutes followed by centrifugation at 15,000 x g for 10 minutes. Supernatant (300 µL) was dried in a nitrogen blower and reconstituted in water:methanol (8:2 v:v) for LC-MS analysis. Reverse phase analyses were performed on an Agilent 1290 Infinity II/ 6545 qTOF MS system with a Jetstream ESI source (Agilent Technologies) using a Waters Acquity HSS T3 column (Waters). Each sample was analyzed in both the positive and negative mode. Ion pairing analyses were performed on an identically-configured LC-MS system with an Agilent Zorbax Extend C18 1.8 µm RRHD column equipped with a matched guard column. Mass spectrometry analysis was conducted in the negative ion mode. Profinder (v8.0) software (Agilent) was used for targeted compound detection and relative quantitation, while custom scripts were used for non-targeted feature detection. Agilent Mass Profiler Pro (v8.0) and Masshunter Qualitative Analysis were used for feature alignment and recursive feature detection. Features with > 50% missing values across samples in a batch or > 30% missing values in QC samples were removed, after which data reduction was performed using Binner[5] and normalized using the "Systematic Error Removal Using Random Forest" approach[6].

Untargeted lipidomics

For untargeted lipidomics analysis, 10 mg of tissue was homogenized in 400 µL isopropanol containing stable isotope-labeled internal standards from Avanti Polar Lipids (Alabaster) using freeze-thaw cycles in liquid nitrogen and sonication. Samples were centrifuged for 5 minutes at 21,000 x g and supernatants were used for LC-MS on a Vanquish chromatography system with an Accucore C30 column (2.1 x 150 mm, 2.6 µm particle size) coupled to a Q-Exactive HF mass spectrometer (ThermoFisher Scientific). Full details on elution gradients and instrument settings are provided elsewhere[1]. Raw LC-MS data was processed with Compound Discoverer v3.0 (ThermoFisher Scientific). Peak area was corrected for QC sample peak area across the batch and filtered with background and QC filters. Features absent in > 50% of the QC pooled injections with a coefficient of variation < 30% were removed from the dataset. Feature annotation was based on mass and relative abundance, retention time and MS2 patterns.

Targeted metabolomics and lipidomics

Branched-chain keto acids, acyl-CoAs and nucleotides were measured by targeted assays at Duke University. For analysis of branched-chain keto acids, 10 µL of plasma or 200 µL of tissue homogenate was extracted using ethyl acetate as previously described[7]. For Acyl-CoA extraction, 500 µL tissue homogenate was used as reported previously for liquid[8] and solid[9] phase extractions. Nucleotides were extracted as previously described[10,11]. Extractions were centrifuged at 14,000 x g for 5 minutes, and supernatants were analyzed by LC-MS/MS using a Xevo TQ-S triple quadrupole mass spectrometer (Waters). Endogenous levels were quantified by spiking tissue homogenates (acyl-coAs and nucleotides) or fetal bovine serum (keto acids) with authentic analytes (Sigma-Aldrich).

Amino acids and amino metabolites, TCA cycle metabolites, ceramides, and acylcarnitines were measured by targeted assays at the Mayo Clinic. For amino acid and amino metabolites, 5 mg of tissue homogenate was extracted and analyzed as previously described[12,13]. Ceramides and sphingolipids were extracted from 5 mg of tissue homogenate as previously described[14,15]; acylcarnitines were also extracted from 5 mg using previously described methods[16,17]. TCA metabolites were extracted from 5 mg of tissue and quantified using GC-MS[18], with minor modifications as reported elsewhere[1].

Targeted lipidomics analysis was performed at Emory University using 10 mg of cryopulverized tissue, homogenized in 100 µL PBS and diluted with 100 µL 20% methanol and spiked with 1% BHT solution according to previously used methods[19,20]. Homogenates were centrifuged at 14,000 x g for 10 minutes and supernatants were loaded onto C18 SPE columns and eluted with 400 µL methyl formate. External standards were purchased from Cayman Chemical. Samples were analyzed by LC-MS/MS using an ExionLC (SCIEX™) chromatography system equipped with an Accucore™ C18 column (ThermoFisher) coupled to a SCIEX™ QTRAP 5500 mass spectrometer. Mobile phase A was water with 10 mM ammonium acetate, and mobile phase B was acetonitrile with 10 mM ammonium acetate. Details on the gradient program and subsequent mass spectrometry analysis are provided elsewhere[1]; raw data was processed using SCIEX™ OS (AB SCIEX™, v1.6.1).

Metabolomics data processing and normalization

All metabolomics datasets were partitioned into named compounds (for analytes that were confidently identified) and unnamed compounds (for those without a standard chemical name). Only named metabolites were included in this analysis. Data was $\log_2$-transformed, and analytes with > 20% missing values were removed. For targeted datasets with > 12 analytes, and for all untargeted datasets, missing values were imputed using K-nearest neighbors (k = 10). Median sample–sample correlation was used to identify outlier samples, which were manually reviewed by the metabolomics sites. Untargeted datasets were normalized using sample median-centering.

Due to the overlap in the coverage of different metabolomics and lipidomics assays, some metabolites/lipids were measured in multiple platforms. Redundant annotated features were defined as the metabolites/lipids that share the same annotation or the same standardized name according to the Metabolomics Workbench RefMet database[21]. Of the 1286 annotated metabolites/lipids, 353 were identified as redundant (i.e., measured in multiple platforms), representing 144 unique metabolites/lipids. These redundant metabolites/lipids were curated by taking into consideration their properties (polarity, solubility, etc.) and their respective assay methodologies (extraction solvent, elution solvent, column, etc.). The exact curation steps are as follows:

1. Internal standards were removed.
2. Measures from targeted assays were selected over untargeted assays.
3. For lipids (3 LPCs, 4 PCs) measured by HILIC and reverse-phase metabolomics, reverse-phase was selected.

4. For lipids that were measured by both the positive and negative modes within the untargeted lipidomics assays, positive mode measurements were selected for lipids that contain a positively charged head group: PC, PE, LPC, LPE, SM.
5. For lipids measured by both positive and negative modes within the untargeted lipidomics assay, or by both positive and negative modes within reverse-phase metabolomics assay, negative mode measurements were selected for lipids that contain a negatively charged head group: phosphatidylinositol (PI), phosphatidylserine (PS), phosphatidic acid (PA), and free fatty acids (FFA).
6. For acylcarnitines with ≤ 12 carbons, HILIC was selected over reverse-phase metabolomics or untargeted lipidomics; for acylcarnitines with > 12 carbons, the selection was made according to the following hierarchy: untargeted lipidomics > HILIC > reverse-phase metabolomics.
7. For SMs, untargeted lipidomics was selected over reverse-phase metabolomics, and positive mode was selected over negative mode within untargeted lipidomics. That is, positive untargeted lipidomics > negative untargeted lipidomics > reverse-phase metabolomics.
8. The rest of the features were visually inspected and selection was made case-by-case.

A detailed list of the 353 redundant metabolites/lipids can be found in Supplementary Table 9.

Software

Statistical Analyses

The R programming language[4] was used to perform all statistical analyses and generate most figures. The following R/Bioconductor[22] packages formed the core of what was used: *ComplexHeatmap*[23] (v2.12.0), *edgeR*[24] (v3.40.1), *emmeans*[25] (v1.8.5), *fgsea*[26] (v1.26.0), *limma*[3] (v3.54.0), *msigdbr*[27] (v7.5.1), *tidyverse*[28] (v2.0.0), and *WGCNA*[29] (v.1.71).

Data and analysis tools for the MoTrPAC landscape paper[1] are also provided through the *MotracRatTraining6moData*[30] and *MotrpacRatTraining6mo*[31] R packages, respectively (github.com/MoTrPAC/MotrpacRatTraining6moData, github.com/MoTrPAC/MotrpacRatTraining6mo); the former package was used to access data for *MotrpacRatTraining6moWATData* (github.com/PNNL-Comp-Mass-Spec/MotrpacRatTraining6moWATData).

Gene Set Network Diagram

We utilized Cytoscape (v3.9.1)[32] to generate a network diagram (Fig. 2D) of the top significantly enriched biological processes (BH-adjusted[33] p-value < 0.05) from the SED male versus SED female proteomics FGSEA results. The combined Jaccard and overlap coefficient[34] was used to calculate edge weights, and only edge weights ≥ 0.25 were retained. Clusters of nodes were manually assigned summary descriptors, and only the top enriched groups are shown.

mTOR Diagram

Figure 3F was created with BioRender.com (www.biorender.com). The leading edge phosphosites were mapped to the human reference phosphoproteome[1] and indicated in pink.

Statistical Analyses

Differential Analysis

While -omics analyses were only performed for 5–6 animals per experimental group, limma does not partition the data into subsets to perform comparisons[3], so the residual degrees of freedom (d.f.) for each feature are (60 samples - 10 experimental groups) = 50 d.f. for proteomics and phosphoproteomics, (50 samples - 2 outliers - 10 groups - 4 covariates) = 34 d.f. for transcriptomics, and (50 samples - 10 groups) = 40 d.f. for metabolomics. Additionally, limma moderates the standard errors of the estimated fold-changes by shrinking them toward a common value or a global trend. The resulting moderated t-statistics follow t-distributions with augmented degrees of freedom, thereby increasing stability and power to detect differences[35,36]. This is further improved by our use of sample quality weights[37] and choice of inbred F344 rats as the model organism—we expect less within-group variation than if we had used outbred rats.

Fast Gene Set Enrichment Analysis

FGSEA was performed with *fgsea::fgseaMultiLevel* (v1.24.0)[26] in R. This employs an adaptive multi-level split Monte Carlo scheme for the estimation of arbitrarily small p-values[26]. GSEA and, by extension, FGSEA, is especially useful when too few or too many genes meet the threshold for statistical significance after correcting for multiple hypothesis testing[38]. The ranking metric we chose is similar to the moderated t-statistic, but the values are more readily interpretable (e.g. a value of 2 means a positive mean difference with a p-value of $10^{-2} = 0.01$).

A total of 10,000 permutations were used for the preliminary estimation of enrichment p-values and to calculate normalized enrichment scores (NES), and p-values were adjusted across groups of related contrasts within each -ome and feature set collection (e.g., across all proteomics GO:MF results) using the BH procedure[33]. The enrichment heatmaps (Figs. 3, S3, S5) display the terms with the highest -$\log_{10}$-transformed adjusted p-value in any contrast. The circles are scaled such that the lowest adjusted p-value in each row is of maximum area; this is to prevent one or more feature sets with extremely low adjusted p-values from dominating the heatmaps. NES in [-1, 1] are essentially noise[26,38], so those circles are not shown.

The MSigDB C5:GO collections were chosen, in part, because they have been filtered to reduce inter-set redundancy using a method similar to the one described by Liberzon *et al*.[39]; the exact method is outlined in sections 3.2 and 3.7 of the v7.0 MSigDB Release Notes on the Broad Institute's website (https://software.broadinstitute.org/cancer/software/gsea/wiki/index.php/MSigDB_v7.0_Release_Notes). After filtering each set to the genes from a particular -ome, they were required to have retained at least 85% of their original members and to contain at least 15 and no more than 300 genes. The high membership percentage cutoff was imposed due to the high proteome (9964 proteins) and transcriptome (16404 transcripts) coverage that was observed for white adipose tissue. The same 85% membership filter was applied to the MitoCarta 3.0 sets, though they

were only required to contain at least 5 and no more than 300 genes due to their already small sizes; this resulted in 68 sets for testing. Since the RefMet chemical subclasses are disjoint and homogenous, they were not filtered based on a membership percentage, though each subclass was required to contain at least 10 metabolites. Set size was restricted because smaller sets are less reliable (more variable) while larger sets are less interpretable.

Kinase–Substrate Enrichment Analysis

As with previous FGSEA, a total of 10,000 permutations were used for the preliminary estimation of KSEA p-values and NES, and p-values were adjusted across all kinases with the BH procedure[33].

The KSEA approach discussed in the Methods is similar to that of Ochoa *et al.*[40], though with several notable improvements owing to the more sophisticated FGSEA algorithm and up-to-date kinase–substrate relationship data. Namely, enrichment p-values are now based on normalized enrichment scores, which account for differences in feature set size, and they are no longer bounded by 0.001 (requiring at least 1/1000 permutation scores to be more extreme). Furthermore, we are able to impose limits on set size to exclude smaller, less reliable sets. This analysis is still limited by the coverage of PSP, however, as the kinases of only 1,074 of the 19,173 phosphosites were known, and only 121 out of a possible 379 kinases passed the size filter for testing.

Weighted Gene Co-expression Network Analysis

Signed adjacency matrices were constructed from the pairwise protein, metabolite, or transcript biweight midcorrelations using a soft-thresholding power of 25 for transcriptomics and 12 for both proteomics and metabolomics/lipidomics. Additionally, transcriptomics count data was converted to $\log_2$ TMM-normalized counts per million reads with *edgeR::cpm*, per the workflow described by Law *et al.*[41] (Differential Analysis Methods). No other processing was performed prior to WGCNA.

Average linkage hierarchical clustering was performed on the dissimilarity matrices–computed from unsigned topological overlap matrices–to define the protein, metabolite/lipid, and transcript modules. A total of 6 metabolites and 2587 transcripts were not co-expressed (assigned to “grey” modules) and subsequently discarded. The remaining 1057 metabolites/lipids were assigned to 7 modules of size 30 to 415, the 9964 quantified proteins to 7 modules of size 165 to 3984, and the 13817 transcripts to 14 modules of size 33 to 4683.

For an in-depth overview of WGCNA, including descriptions of biweight midcorrelation and module eigengenes (which we more generally refer to as eigenfeatures), we recommend Weighted Network Analysis: Applications in Genomics and Systems Biology[42].

Spearman correlations between metabolomics/lipidomics and other -omics MEs and between the MEs and select sample measures, as well as their significance, were assessed with *stats::cor.test*[4]. P-values were adjusted within each individual heatmap using the BH procedure[33] to control the FDR.

WGCNA Module Over-Representation Analysis

Since the p-value histograms resulting from ORA exhibited peaks near both 0 and 1 (the latter indicating under-represented sets), and there appeared to be an association between p-value and feature set size, p-values were not adjusted to control the FDR. Instead, an overlap ratio (equation (1)) was calculated by dividing the cardinality of the intersection between each module and feature set by one plus the cardinality of the module-wise maximum intersection. (Adding 1 to the denominator penalizes small maximum intersections.)

$$\text{overlap ratio}_{ij} = \frac{|\text{module}_i \cap \text{feature set}_j|}{max_k(|\text{module}_i \cap \text{feature set}_k|) + 1} \qquad (1)$$

The over-representation p-values were then $\log_{10}$-transformed, multiplied by their associated overlap ratio, and back-transformed. This removed the association between set size and p-value, and the histograms of these scaled p-values appeared left-skewed. A small scaled p-value indicates that a feature set is over-represented in a particular module and explains it well relative to all terms that were tested.

References

1. MoTrPAC Study Group *et al.* Temporal dynamics of the multi-omic response to endurance exercise training across tissues. *bioRxiv* 2022.09.21.508770 (2022) doi:10.1101/2022.09.21.508770.
2. Mertins, P. *et al.* Reproducible workflow for multiplexed deep-scale proteome and phosphoproteome analysis of tumor tissues by liquid chromatography-mass spectrometry. *Nat. Protoc.* **13**, 1632–1661 (2018).
3. Ritchie, M. E. *et al.* limma powers differential expression analyses for RNA-sequencing and microarray studies. *Nucleic Acids Res.* **43**, e47 (2015).
4. R Core Team. R: A Language and Environment for Statistical Computing. Preprint at https://www.R-project.org/ (2021).
5. Kachman, M. *et al.* Deep annotation of untargeted LC-MS metabolomics data with Binner. *Bioinformatics* **36**, 1801–1806 (2020).
6. Fan, S. *et al.* Systematic Error Removal Using Random Forest for Normalizing Large-Scale Untargeted Lipidomics Data. *Anal. Chem.* **91**, 3590–3596 (2019).
7. Kwee, L. C. *et al.* Metabolites and diabetes remission after weight loss. *Nutr. Diabetes* **11**, 10 (2021).
8. Magnes, C., Sinner, F. M., Regittnig, W. & Pieber, T. R. LC/MS/MS method for quantitative determination of long-chain fatty acyl-CoAs. *Anal. Chem.* **77**, 2889–2894 (2005).
9. Minkler, P. E., Kerner, J., Ingalls, S. T. & Hoppel, C. L. Novel isolation procedure for short-, medium-, and long-chain acyl-coenzyme A esters from tissue. *Anal. Biochem.* **376**, 275–276 (2008).
10. Cordell, R. L., Hill, S. J., Ortori, C. A. & Barrett, D. A. Quantitative profiling of nucleotides and related phosphate-containing metabolites in cultured mammalian cells by liquid chromatography tandem electrospray mass spectrometry. *J. Chromatogr. B Analyt. Technol. Biomed. Life Sci.* **871**, 115–124 (2008).

11. Gooding, J. R. *et al.* Adenylosuccinate Is an Insulin Secretagogue Derived from Glucose-Induced Purine Metabolism. *Cell Rep.* **13**, 157–167 (2015).

12. Gonsalves, W. I. *et al.* Metabolomic and Lipidomic Profiling of Bone Marrow Plasma Differentiates Patients with Monoclonal Gammopathy of Undetermined Significance from Multiple Myeloma. *Sci. Rep.* **10**, 10250 (2020).

13. Lanza, I. R. *et al.* Quantitative metabolomics by H-NMR and LC-MS/MS confirms altered metabolic pathways in diabetes. *PLoS One* **5**, e10538 (2010).

14. Blachnio-Zabielska, A. U., Persson, X.-M. T., Koutsari, C., Zabielski, P. & Jensen, M. D. A liquid chromatography/tandem mass spectrometry method for measuring the in vivo incorporation of plasma free fatty acids into intramyocellular ceramides in humans. *Rapid Commun. Mass Spectrom.* **26**, 1134–1140 (2012).

15. Savica, R. *et al.* Plasma sphingolipid changes with autopsy-confirmed Lewy Body or Alzheimer's pathology. *Alzheimers. Dement.* **3**, 43–50 (2016).

16. Chace, D. H. *et al.* Electrospray tandem mass spectrometry for analysis of acylcarnitines in dried postmortem blood specimens collected at autopsy from infants with unexplained cause of death. *Clin. Chem.* **47**, 1166–1182 (2001).

17. Wilkins, J., Sakrikar, D., Petterson, X.-M., Lanza, I. R. & Trushina, E. A comprehensive protocol for multiplatform metabolomics analysis in patient-derived skin fibroblasts. *Metabolomics* **15**, 83 (2019).

18. Dutta, T. *et al.* Impact of Long-Term Poor and Good Glycemic Control on Metabolomics Alterations in Type 1 Diabetic People. *J. Clin. Endocrinol. Metab.* **101**, 1023–1033 (2016).

19. Maner-Smith, K. M. *et al.* Alterations in the Human Plasma Lipidome in Response to Tularemia Vaccination. *Vaccines (Basel)* **8**, (2020).

20. Collins, J. M. *et al.* TCA cycle remodeling drives proinflammatory signaling in humans with pulmonary tuberculosis. *PLoS Pathog.* **17**, e1009941 (2021).

21. Fahy, E. & Subramaniam, S. RefMet: a reference nomenclature for metabolomics. *Nat.*

Methods **17**, 1173–1174 (2020).

22. Huber *et al.* Orchestrating high-throughput genomic analysis with Bioconductor. *Nature methods* Preprint at http://www.nature.com/nmeth/journal/v12/n2/full/nmeth.3252.html.
23. Gu, Z., Eils, R. & Schlesner, M. Complex heatmaps reveal patterns and correlations in multidimensional genomic data. *Bioinformatics* **32**, 2847–2849 (2016).
24. Robinson, M. D., McCarthy, D. J. & Smyth, G. K. edgeR: a Bioconductor package for differential expression analysis of digital gene expression data. *Bioinformatics* **26**, 139–140 (2010).
25. Lenth, R. V. emmeans: Estimated Marginal Means, aka Least-Squares Means. Preprint at https://CRAN.R-project.org/package=emmeans (2023).
26. Korotkevich, G. *et al.* Fast gene set enrichment analysis. *bioRxiv* 060012 (2021) doi:10.1101/060012.
27. Dolgalev, I. msigdbr: MSigDB Gene Sets for Multiple Organisms in a Tidy Data Format. Preprint at https://CRAN.R-project.org/package=msigdbr (2022).
28. Wickham, H. *et al.* Welcome to the tidyverse. *Journal of Open Source Software* vol. 4 1686 Preprint at https://doi.org/10.21105/joss.01686 (2019).
29. Langfelder, P. & Horvath, S. WGCNA: an R package for weighted correlation network analysis. *BMC Bioinformatics* **9**, 559 (2008).
30. Gay, N., Jean Beltran, P., Amar, D., Jimenez-Morales, D. & MoTrPAC Study Group. MotrpacRatTraining6moData: Data for analysis of the MoTrPAC endurance exercise training study in 6-month-old rats. Preprint at (2022).
31. Gay, N., Amar, D., Jean Beltran, P. & MoTrPAC Study Group. MotrpacRatTraining6mo: Analysis of the MoTrPAC endurance exercise training data in 6-month-old rats. Preprint at (2023).
32. Shannon, P. *et al.* Cytoscape: a software environment for integrated models of biomolecular interaction networks. *Genome Res.* **13**, 2498–2504 (2003).

33. Benjamini, Y. & Hochberg, Y. Controlling the False Discovery Rate: A Practical and Powerful Approach to Multiple Testing. *Journal of the Royal Statistical Society: Series B (Methodological)* vol. 57 289–300 Preprint at https://doi.org/10.1111/j.2517-6161.1995.tb02031.x (1995).

34. Isserlin, R., Merico, D., Voisin, V. & Bader, G. D. Enrichment Map - a Cytoscape app to visualize and explore OMICs pathway enrichment results. *F1000Res.* **3**, 141 (2014).

35. Smyth, G. K. Linear models and empirical bayes methods for assessing differential expression in microarray experiments. *Stat. Appl. Genet. Mol. Biol.* **3**, Article3 (2004).

36. Phipson, B., Lee, S., Majewski, I. J., Alexander, W. S. & Smyth, G. K. ROBUST HYPERPARAMETER ESTIMATION PROTECTS AGAINST HYPERVARIABLE GENES AND IMPROVES POWER TO DETECT DIFFERENTIAL EXPRESSION. *Ann. Appl. Stat.* **10**, 946–963 (2016).

37. Liu, R. *et al.* Why weight? Modelling sample and observational level variability improves power in RNA-seq analyses. *Nucleic Acids Res.* **43**, e97 (2015).

38. Subramanian, A. *et al.* Gene set enrichment analysis: a knowledge-based approach for interpreting genome-wide expression profiles. *Proc. Natl. Acad. Sci. U. S. A.* **102**, 15545–15550 (2005).

39. Liberzon, A. *et al.* The Molecular Signatures Database (MSigDB) hallmark gene set collection. *Cell Syst* **1**, 417–425 (2015).

40. Ochoa, D. *et al.* An atlas of human kinase regulation. *Mol. Syst. Biol.* **12**, 888 (2016).

41. Law, C. W. *et al.* RNA-seq analysis is easy as 1-2-3 with limma, Glimma and edgeR. *F1000Res.* **5**, (2016).

42. Horvath, S. *Weighted Network Analysis: Applications in Genomics and Systems Biology*. (Springer New York, 2011).
