# Supplementary material for: Sexual dimorphism and the multi-omic response to exercise training in rat subcutaneous white adipose tissue
Source: Nat Metab. 2024 May 1;6(5):963–79. doi: 10.1038/s42255-023-00959-9 (PMC11132991; doi:10.1038/s42255-023-00959-9)
Supplement: Supplementary file 2 — Reporting Summary [file 42255_2023_959_MOESM2_ESM.pdf]

nature portfolio

Corresponding author(s): Joshua N. Adkins, Sue C. Bodine, Christopher B. Newgard, Simon Schenk

Last updated by author(s): Jan 16, 2024

Reporting Summary

Nature Portfolio wishes to improve the reproducibility of the work that we publish. This form provides structure for consistency and transparency in reporting. For further information on Nature Portfolio policies, see our Editorial Policies and the Editorial Policy Checklist.

Statistics

For all statistical analyses, confirm that the following items are present in the figure legend, table legend, main text, or Methods section.

n/a	Confirmed	
☐	☒	The exact sample size (*n*) for each experimental group/condition, given as a discrete number and unit of measurement
☐	☒	A statement on whether measurements were taken from distinct samples or whether the same sample was measured repeatedly
☐	☒	The statistical test(s) used AND whether they are one- or two-sided<br>*Only common tests should be described solely by name; describe more complex techniques in the Methods section.*
☐	☒	A description of all covariates tested
☐	☒	A description of any assumptions or corrections, such as tests of normality and adjustment for multiple comparisons
☐	☒	A full description of the statistical parameters including central tendency (e.g. means) or other basic estimates (e.g. regression coefficient) AND variation (e.g. standard deviation) or associated estimates of uncertainty (e.g. confidence intervals)
☐	☒	For null hypothesis testing, the test statistic (e.g. *F*, *t*, *r*) with confidence intervals, effect sizes, degrees of freedom and *P* value noted<br>*Give P values as exact values whenever suitable.*
☒	☐	For Bayesian analysis, information on the choice of priors and Markov chain Monte Carlo settings
☒	☐	For hierarchical and complex designs, identification of the appropriate level for tests and full reporting of outcomes
☐	☒	Estimates of effect sizes (e.g. Cohen's *d*, Pearson's *r*), indicating how they were calculated

Our web collection on statistics for biologists contains articles on many of the points above.

Software and code

Policy information about availability of computer code

Data collection	Software sources and versions used for multi-omic data collection are specified in the Methods and Supplementary Methods included with this manuscript.
Data analysis	Software sources and versions used for data analysis are specified in the Methods and Supplementary Methods. The R programming language was used to perform all statistical analyses, utilizing the following R/Bioconductor packages: ComplexHeatmap (v2.12.0), edgeR (v3.40.1), emmeans (v1.8.5), fgsea (v1.26.0), limma (v3.54.0), msigdbr (v7.5.1), tidyverse (v2.0.0) and WGCNA (v1.7.1). Custom code for reproducing the main analyses are conveniently provided in the MotrpacRatTraining6moWAT R package (github.com/MoTrPAC/MotrpacRatTraining6moWAT) and described in the "Code Availability" section of the manuscript. Data and analysis tools for the MoTrPAC landscape paper from wihch these datasets were accessed (https://www.biorxiv.org/content/10.1101/2022.09.21.508770v3) are also provided through the MotracRatTraining6moData and MotrpacRatTraining6mo R packages, respectively (github.com/MoTrPAC/MotrpacRatTraining6moData, github.com/MoTrPAC/MotrpacRatTraining6mo).

For manuscripts utilizing custom algorithms or software that are central to the research but not yet described in published literature, software must be made available to editors and reviewers. We strongly encourage code deposition in a community repository (e.g. GitHub). See the Nature Portfolio guidelines for submitting code & software for further information.

Data

Policy information about availability of data

All manuscripts must include a data availability statement. This statement should provide the following information, where applicable:

- Accession codes, unique identifiers, or web links for publicly available datasets
- A description of any restrictions on data availability
- For clinical datasets or third party data, please ensure that the statement adheres to our policy

The final version of this manuscript provides all relevant information in the "Data Availability" section. Processed data used for/ analysis results generated in this manuscript are available in the MotrpacRatTraining6moWATData R package (github.com/MoTrPAC/MotrpacRatTraining6moWATData). MoTrPAC data related to this study, but not used in this manuscript, is publicly available via motrpac-data.org, with no registration or login requirement for data access; additional resources can be found at motrpac.org and motrpac-data.org. The following external datasets were used for this manuscript: MitoCarta3.0 (Human.MitoCarta3.0.xls, accessed from https://www.broadinstitute.org/mitocarta/mitocarta30-inventory-mammalian-mitochondrial-proteins-and-pathways); PhosophositePlus (v6.6.0.4; Kinase_Substrate_Dataset.xlsx, accessed 2022-06-05 from https://www.phosphosite.org/staticDownloads); MSigDB (v7.5.1, https://www.gsea-msigdb.org/gsea/msigdb); and the Metabolomics Workbench RefMet database (https://www.metabolomicsworkbench.org). Raw and processed transcriptomics, metabolomics, and proteomics data have been deposited in public repositories (GEO, Metabolomics Workbench, and MassIVE, respectively), with the relevant accession codes and links provided in the "Data Availability" section of this manuscript.

Human research participants

Policy information about studies involving human research participants and Sex and Gender in Research.

Reporting on sex and gender	N/A - no human participants
Population characteristics	N/A - no human participants
Recruitment	N/A - no human participants
Ethics oversight	N/A - no human participants

Note that full information on the approval of the study protocol must also be provided in the manuscript.

Field-specific reporting

Please select the one below that is the best fit for your research. If you are not sure, read the appropriate sections before making your selection.

☒ Life sciences ☐ Behavioural & social sciences ☐ Ecological, evolutionary & environmental sciences

For a reference copy of the document with all sections, see nature.com/documents/nr-reporting-summary-flat.pdf

Life sciences study design

All studies must disclose on these points even when the disclosure is negative.

Sample size	Experimental groups consisted of n=5 biological replicates per sex/group for metabolomics/lipidomics, transcriptomics, and mtDNA quantification, and n=6 biological replicates per sex/group for proteomics/phosphoproteomics, clinical analyte measures in plasma, histology, and phenotypic measures. No statistical methods were used to predetermine sample sizes; these sample size decisions were dictated by a combination of resource availability and assay-specific expertise, and are consistent with what has been used in previous rodent studies (Emont, M.P. et al., Nature 2022; Stanford, K.I. et al., Diabetes 2015; Macotela, Y. et al., Diabetes 2009; Xiong, Y. et al., J Lipid Res 2018; Levt, J. et al., Am J Physiol Endocrinol Metab 2000).
Data exclusions	Data used in this manuscript was accessed through the MotrpacRatTraining6moData R package (https://motrpac.github.io/MotrpacRatTraining6moData/). Outlier samples had already been removed based on methods described in the Landscape paper associated with this study (https://www.biorxiv.org/content/10.1101/2022.09.21.508770v3) which has been provisionally accepted in Nature.
Replication	5-6 biological replicates of each sex were used in each experimental group. Given the scale of the data and the goal of providing a data resource for hypothesis generation, we did not attempt to reproduce our findings.
Randomization	Following an initial acclimation period, rats went through a 12-day treadmill familiarization protocol to expose the rats to the treadmill and to identify potential non-compliant rats. Those rats that were unable to run on the treadmill for 5 minutes at a speed of 10 m/min and grade of 0° were classified as non-compliant and removed from the study. Rats that successfully completed the 12-day familiarization protocol were entered in the rat database and randomized into a control or training group so that mean body weight of the groups were equal. The 8-week rats were randomly assigned to control or training within sex and tertile of weight. 4-week rats were assigned to control without randomization. 1- and 2- week rats were randomly assigned to 1- or 2-week training within sex and tertile of weight.
Blinding	At all multi-omic analysis sites, an unblinded batching officer was responsible for randomization of the samples across batches of appropriate

Blinding	size for the analysis platforms in place, using experimental data to avoid confounding between batch and experimental groups. Randomized samples were blinded to all individuals involved in sample preparation, data generation, and initial data processing. Downstream quality control and data analysis were not performed blind to the conditions of the experiments, to allow for the appropriate comparisons to be tested by the statistical models employed.

Reporting for specific materials, systems and methods

We require information from authors about some types of materials, experimental systems and methods used in many studies. Here, indicate whether each material, system or method listed is relevant to your study. If you are not sure if a list item applies to your research, read the appropriate section before selecting a response.

Materials & experimental systems

n/a	Involved in the study	
☒	☐	Antibodies
☒	☐	Eukaryotic cell lines
☒	☐	Palaeontology and archaeology
☐	☒	Animals and other organisms
☒	☐	Clinical data
☒	☐	Dual use research of concern

Methods

n/a	Involved in the study	
☒	☐	ChIP-seq
☒	☐	Flow cytometry
☒	☐	MRI-based neuroimaging

Animals and other research organisms

Policy information about studies involving animals; ARRIVE guidelines recommended for reporting animal research, and Sex and Gender in Research

Laboratory animals	Adult male and female Fischer 344 (F344) inbred rats were obtained from the National Institute on Aging (NIA) rodent colony. All animals were 6 months old at the beginning of the intervention.
Wild animals	The study did not involve wild animals.
Reporting on sex	Equal numbers of male and female animals were included in the study. Sex-stratified results are described extensively.
Field-collected samples	The study did not involve samples collected from the field.
Ethics oversight	All animal procedures were approved by the Institutional Animal Care and Use Committee at the University of Iowa.

Note that full information on the approval of the study protocol must also be provided in the manuscript.
