# Supplementary figures and images for: Sexual dimorphism and the multi-omic response to exercise training in rat subcutaneous white adipose tissue
Source: Nat Metab. 2024 May 1;6(5):963–79. doi: 10.1038/s42255-023-00959-9 (PMC11132991; doi:10.1038/s42255-023-00959-9)

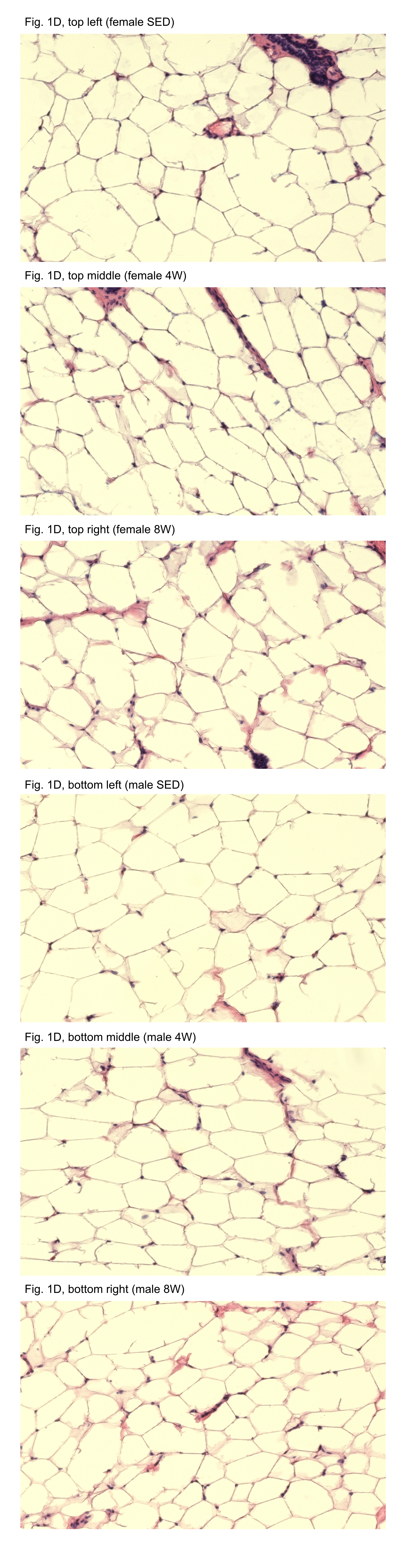

Supplement: Supplementary file 4 — Unprocessed microscopy image for Fig. 1d. [file 42255_2023_959_MOESM4_ESM.jpg]
